# A formamidopyrimidine derivative from the deoxyguanosine adduct produced by food contaminant acrylamide induces DNA replication block and mutagenesis

**DOI:** 10.1016/j.jbc.2023.105002

**Published:** 2023-06-30

**Authors:** Jun-ichi Akagi, Masayuki Yokoi, Yumi Miyake, Tsuyoshi Shirai, Tomohiro Baba, Young-Man Cho, Fumio Hanaoka, Kaoru Sugasawa, Shigenori Iwai, Kumiko Ogawa

**Affiliations:** 1 Division of Pathology, National Institute of Health Sciences, Kawasaki, Kanagawa, Japan; 2 Biosignal Research Center, Kobe University, Kobe, Hyogo, Japan; 3 Forefront Research Center, Graduate School of Science, Osaka University, Toyonaka, Osaka, Japan; 4 Department of Bioscience, Nagahama Institute of Bio-Science and Technology, Nagahama, Shiga, Japan; 5 Graduate School of Engineering Science, Osaka University, Toyonaka, Osaka, Japan; 6 National Institute of Genetics, Mishima, Shizuoka, Japan

**Keywords:** DNA damage, mutagenesis, mutagenesis mechanism, DNA replication, nucleic acid chemistry, acrylamide, glycidamide, formamidopyrimidine

## Abstract

Acrylamide, a common food contaminant, is metabolically activated to glycidamide, which reacts with DNA at the N7 position of dG, forming N7-(2-carbamoyl-2-hydroxyethyl)-dG (GA^7^dG). Owing to its chemical lability, the mutagenic potency of GA^7^dG has not yet been clarified. We found that GA^7^dG undergoes ring-opening hydrolysis to form *N*^6^-(2-deoxy-d-*erythro*-pentofuranosyl)-2,6-diamino-3,4-dihydro-4-oxo-5-[*N*-(2-carbamoyl-2-hydroxyethyl)formamido]pyrimidine (GA-FAPy-dG), even at neutral pH. Therefore, we aimed to examine the effects of GA-FAPy-dG on the efficiency and fidelity of DNA replication using an oligonucleotide carrying GA-FAPy–9-(2-deoxy-2-fluoro-β-d-arabinofuranosyl)guanine (dfG), a 2′-fluorine substituted analog of GA-FAPy-dG. GA-FAPy-dfG inhibited primer extension by both human replicative DNA polymerase ε and the translesion DNA synthesis polymerases (Polη, Polι, Polκ, and Polζ) and reduced the replication efficiency by less than half in human cells, with single base substitution at the site of GA-FAPy-dfG. Unlike other formamidopyrimidine derivatives, the most abundant mutation was G:C > A:T transition, which was decreased in Polκ- or REV1-KO cells. Molecular modeling suggested that a 2-carbamoyl-2-hydroxyethyl group at the *N*^5^ position of GA-FAPy-dfG can form an additional H-bond with thymidine, thereby contributing to the mutation. Collectively, our results provide further insight into the mechanisms underlying the mutagenic effects of acrylamide.

Acrylamide is a genotoxic carcinogen classified as group 2A (probably carcinogenic to humans) in the carcinogenicity classification of the International Agency for Research on Cancer (1). Acrylamide is produced by cooking carbohydrate-rich foods at high temperatures (2); it is found in processed foods, such as potato chips, French fries, roasted foods, coffee, corn snacks, and curry powders (3), and is also unintentionally produced by home cooking processes, such as stir-frying, deep-frying, and bread toasting (4). Thus, it is considered an important issue in food safety. The international mean dietary exposures of acrylamide for general and high-exposure populations have been estimated at 1 and 4 μg/kg body weight/day, respectively (5). Doses with a 10% incidence in the upper limit of 95% confidence for carcinogenic effects (BMDL_10_) are 0.18 mg/kg body weight/day for males (Harderian gland adenomas/adenocarcinomas in male mice) (6) and 0.31 mg/kg body weight/day for females (breast fibroadenomas in female rats) (7). Based on these observations, the margins of exposure (MOE), which are calculated by the BMDL_10_ divided by estimated levels of human exposure, are 45 (male high-exposure populations) to 310 (female low-exposure populations) (5). As the MOEs are low for a compound that is genotoxic and carcinogenic, the Joint FAO/WHO Expert Committee on Food Additives considered that these MOEs indicate a human health concern for carcinogenic effects of acrylamide (5).

Acrylamide is metabolized by cytochrome P450 2E1 to glycidamide (GA), a reactive epoxide (8). The carcinogenic activity of acrylamide is believed to be associated with its metabolic conversion to GA (9). GA can also be directly formed in food during cooking (10) and can react with the nucleobases in genomic DNA and other biomolecules. GA mainly reacts with the N7 position of 2′-dG in DNA, resulting in N7-(2-carbamoyl-2-hydroxyethyl)-dG (GA^7^dG) (11). GA can also react with dA; therefore, GA adducts at the N3 position of dA are also found *in vivo*, although this product amounts to less than 1/100th the level of GA^7^dG (12). As the N7-alkylated form of dG and N3-alkylated form of dA are chemically labile and prone to depurination, it has been considered that the resulting apurinic/apyrimidic (AP) sites are responsible for acrylamide and GA-induced mutagenesis (13, 14). However, AP sites are some of the most abundant endogenous DNA lesions, which are generated by spontaneous depurination or by the DNA glycosylase–mediated cleavage of damaged bases and are constantly being repaired. Thus, genomic DNA carries high steady-state levels of AP sites estimated at 8 to 30 lesions per 10^6^ nucleotides (15). Moreover, there is no evidence of an increase in AP sites following acrylamide or GA treatment. Therefore, it is questionable that AP sites derived from the depurination of these GA adducts are the major source of the mutagenic potency of GA. Furthermore, animal studies have indicated that GA^7^dG in genomic DNA persists for at least 24 h after the administration of acrylamide to mice and is accumulated upon repeated administration (16, 17). The discrepancy that GA^7^dG can accumulate in genomic DNA despite its chemical instability may be explained by the decrease in the depurination of N7-alkylated dG in nucleosome core particles (18). However, N7-dG adducts have another inherent lability. Hydrolytic cleavage of the imidazolium ring leads to the formation of *N*^6^-(2-deoxy-d-*erythro*-pentofuranosyl)-2,6-diamino-3,4-dihydro-4-oxo-5-[*N*-(2-carbamoyl-2-hydroxyethyl)formamido]pyrimidine (GA-FAPy-dG), a ring-opened derivative (Fig. 1).

**Figure 1:**
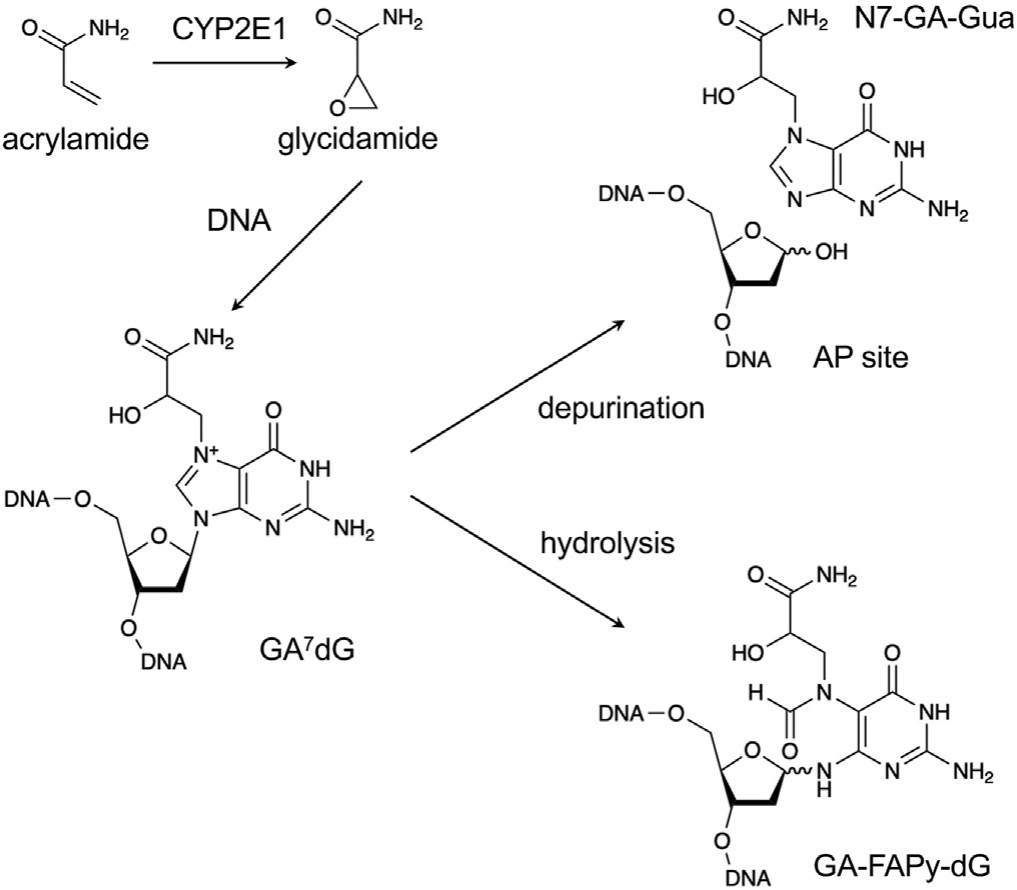
**Formation of GA**^**7**^**dG and its chemical lability.** GA^7^dG, N7-(2-carbamoyl-2-hydroxyethyl)-dG.

Formamidopyrimidine (FAPy) derivatives arise from a number of genotoxic chemicals *via* their N7-dG adducts (19), but their effect on DNA replication and mutagenic mechanisms is poorly understood. Only FAPy-dG (20, 21, 22), methyl (Me)-FAPy-dG (23), and aflatoxin B_1_ (AFB_1_)-FAPy-dG (24) have been studied for their mutagenic potential in mammalian cells. FAPy-dG is produced by oxidative damage in amounts comparable to 8-oxo-dG, but it is more mutagenic than the latter (25). Me-FAPy-dG and AFB_1_-FAPy-dG are more mutagenic than their parental N7-dG adducts and persist in genomic DNA (26). Neutralization of the positively charged N7-dG adducts through ring-opening hydrolysis confers chemical stability to FAPy derivatives. Although accumulation of GA^7^dG following the administration of acrylamide or GA could be quantified as N7-GA-guanine (N7-GA-Gua) released from recovered genomic DNA by neutral thermal hydrolysis, chemically stable GA-FAPy-dG was not detected using the same method. Therefore, the extent to which GA is hydrolyzed to GA-FAPy-dG *in vivo* is unclear; however, chemical stability and mutagenic potential of FAPy derivatives (19, 20, 21, 22, 23, 24, 25, 26) imply that GA-FAPy-dG may contribute to acrylamide-induced mutagenesis.

DNA lesions, such as GA-FAPy-dG, may inhibit the normal DNA replication catalyzed by high-fidelity DNA polymerases (Pol). The inhibition of DNA replication can induce chromosomal instability and lead to cellular catastrophes (27). Translesion DNA synthesis (TLS) is a DNA damage tolerance mechanism that can overcome replication blockages to avoid DNA damage–induced fatalities, by utilizing Pols that have specialized catalytic activities for TLS (TLS Pols) (28). TLS Pols can perform direct replication using DNA lesions as templates; however, as a compensation for damage avoidance, they are prone to incorporate incorrect nucleotides opposite or downstream of the lesions. This results in point mutations, including base substitution mutations and/or small insertions or deletions (29). The *in vivo* and *in vitro* mutagenicity of GA has been reported using reporter gene mutation assays (13, 30, 31, 32, 33, 34), whole exome sequencing (WES) (35), and whole-genome sequencing (WGS) assays (36). However, these assays cannot distinguish whether the mutations originated from persisting GA adducts, AP sites, or the GA-FAPy derivative. Intracellular TLS assays that utilize a shuttle vector carrying a modified nucleotide at the defined positions enable us to discriminate the effects of particular DNA lesions in mammalian cells.

As the major GA-induced DNA damage is the N7-dG adduct, it is likely that GA-FAPy-dG is produced in the same way as other N7-dG alkylating agents. However, the mutagenic potential of GA-FAPy-dG has not been examined. It has been reported that the glycosidic bond of N7-Me-dG can be stabilized using 9-(2-deoxy-2-fluoro-β-d-arabinofuranosyl)guanine (dfG), in which the hydrogen at the 2′ upper position of 2′-deoxyribofuranose is substituted with fluorine, instead of the normal 2′-deoxyribose with no DNA structural distortion (37). On the other hand, the electron-withdrawing substituents on the sugar residue facilitate ring-opening hydrolysis; therefore, the 2′-F isostere approach would allow preferential synthesis of FAPy derivatives. The 2′-F substitution itself apparently affects neither the catalytic activity nor the fidelity of Pols (38, 39, 40, 41). Therefore, we generated a 30-mer oligonucleotide carrying a 2′-F substituted analog of GA-FAPy-dG at a defined position to examine the mutagenic potential of the GA-FAPy derivative in human cells and the contribution of TLS Pols.

## Results

### Reaction of GA with nucleosides

**Figure 2:**
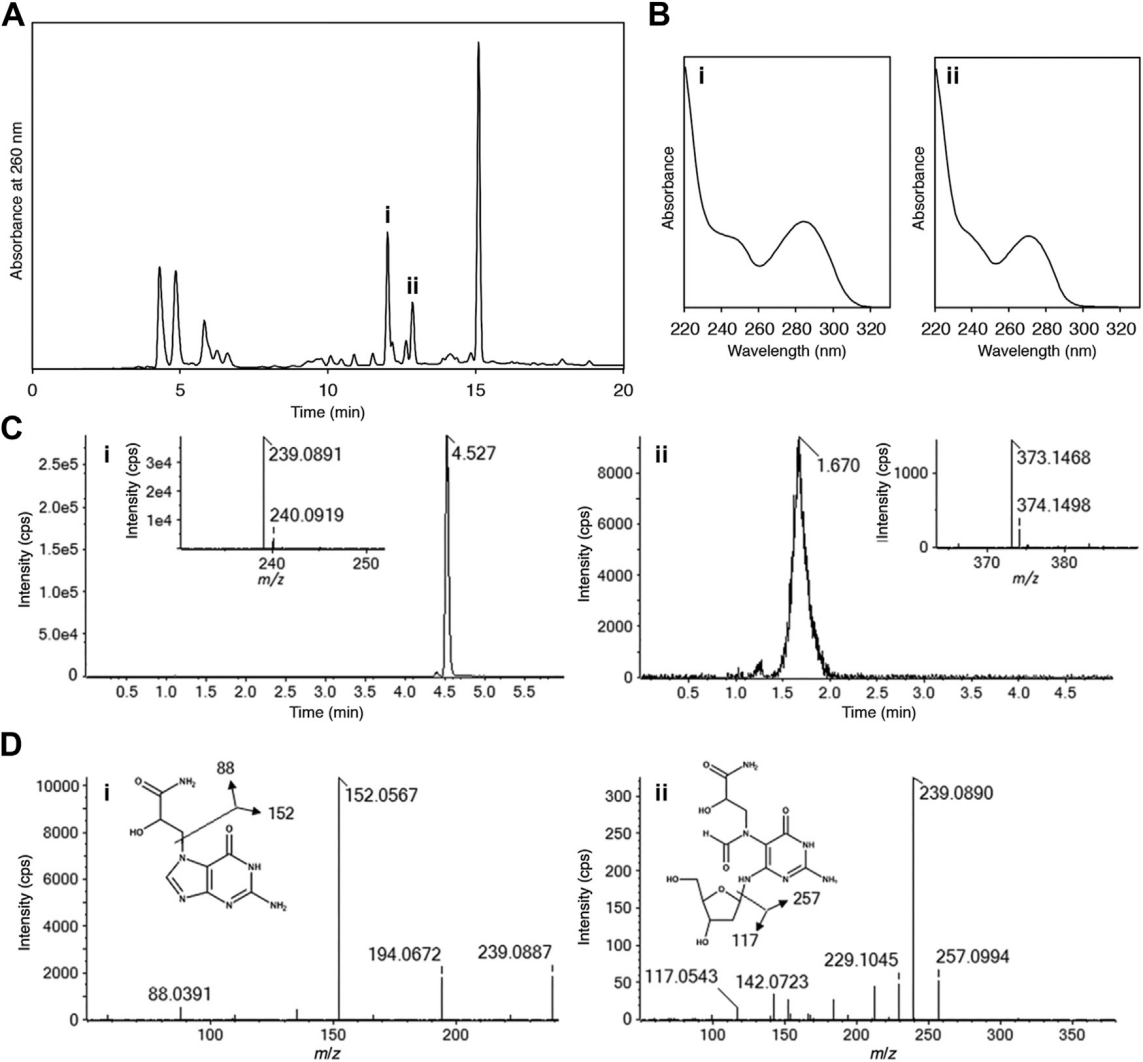
**Detection of GA-FAPy-dG formed in the reaction of dG with GA.** *A*, HPLC analysis of the reaction of dG with GA. The starting material (dG) had a retention time of 15.1 min. *B*, UV absorption spectra of peaks i and ii. *C*, extracted ion chromatogram of peak i and peak ii samples shown in (*A*) analyzed by LC–MS (QTOF-MS). Chromatograms were extracted at *m/z* 239.089 ([M + H]^+^ of N7-GA-Gua]) for peak i sample and at *m/z* 373.147 ([M + H]^+^ of GA-FAPy-dG]) for peak ii sample in 0.01 Da window. The peak i sample was analyzed using a C18 reversed-phase column, and hydrophilic separation was adopted for peak ii sample. Inserted figures show the enlarged mass spectra obtained from peak i (4.527 min) and peak ii (1.670 min). The mass errors of measured *m/z* values were within 1 mDa. *D*, product ion spectra obtained from peaks i and ii, for which the precursor ions were set to *m/z* 239.1 and *m/z* 373.1, respectively. GA, glycidamide; GA-FAPy-dG, *N*^6^-(2-deoxy-d-*erythro*-pentofuranosyl)-2,6-diamino-3,4-dihydro-4-oxo-5-[*N*-(2-carbamoyl-2-hydroxyethyl)formamido]pyrimidine.

To the best of our knowledge, the formation of GA-FAPy-dG has not been reported. Therefore, we tried to detect this product at the nucleoside level. After incubation of dG with GA in a pH 7.0 buffer at 37 °C for 48 h, the reaction mixture was analyzed by reversed-phase HPLC (Fig. 2*A*). The absorption maxima of peaks i and ii were 284.4 and 271.3 nm, respectively (Fig. 2*B*), which are close to those of N7-Me-guanine (282 nm) (42) and Me-FAPy-dG (273 nm) (43). These products were isolated and further analyzed by LC–MS. The peak i sample provided a single peak at 4.527 min in the extracted ion chromatogram of *m/z* 239.089 ([M + H]^+^ of N7-GA-Gua), and the peak ii sample also showed a peak at 1.670 min in the extracted ion chromatogram of *m/z* 373.147 ([M + H]^+^ of GA-FAPy-dG), as shown in Figure 2*C*. The measured *m/z* values obtained from the peaks at 4.527 and 1.670 min were in good agreement with the calculated *m/z* values of [M + H]^+^ of N7-GA-Gua (*m/z* 239.0887) and GA-FAPy-dG (*m/z* 373.1466), respectively. The product ion spectra (tandem mass spectrometry [MS/MS]) obtained from the peak at 4.527 min (precursor ion at *m/z* 239.1) and 1.670 min (precursor ion at *m/z* 373.1) are shown in Figure 2*D*. The collision-induced dissociation of [M + H]^+^ for N7-GA-Gua at *m/z* 239.1 gave the product ions at *m/z* 152.0567 and *m/z* 88.0391, which matched to the formulae of C_5_H_5_N_5_O and C_3_H_5_NO_2_, respectively. The precursor ion for GA-FAPy-dG at *m/z* 373.1 provided the product ions at *m/z* 257.0994 and *m/z* 117.0543, which corresponded to the formulae of C_8_H_12_N_6_O_4_ and C_5_H_8_O_3_, respectively, in the MS/MS analysis. The mass errors of measured *m/z* values for these product ions were within 1 mDa of the calculated *m/z* values. These results indicated that the FAPy product was obtained in the reaction of the guanine base moiety with GA. The molar ratio of the compounds corresponding to peaks i and ii, considering their molar extinction coefficients, was confirmed to be 5.9:1. However, the peaks emerged at 4.30 and 4.85 min exhibited an *m/z* value of 326.1196, corresponding to guanine adducts with two GA molecules (*m/z* 326.1207). Although the molecular structures of these peaks were unknown, they shared the same UV absorption spectrum, indicating that they were isomers of depurinated guanine adducts with two GA molecules. Considering these alternative forms of depurinated GA adduct, the estimated molar ratio of AP sites to GA-FAPy-dG was 16.5:1.

### Preparation of the oligonucleotide containing the GA-FAPy adduct

**Figure 3:**
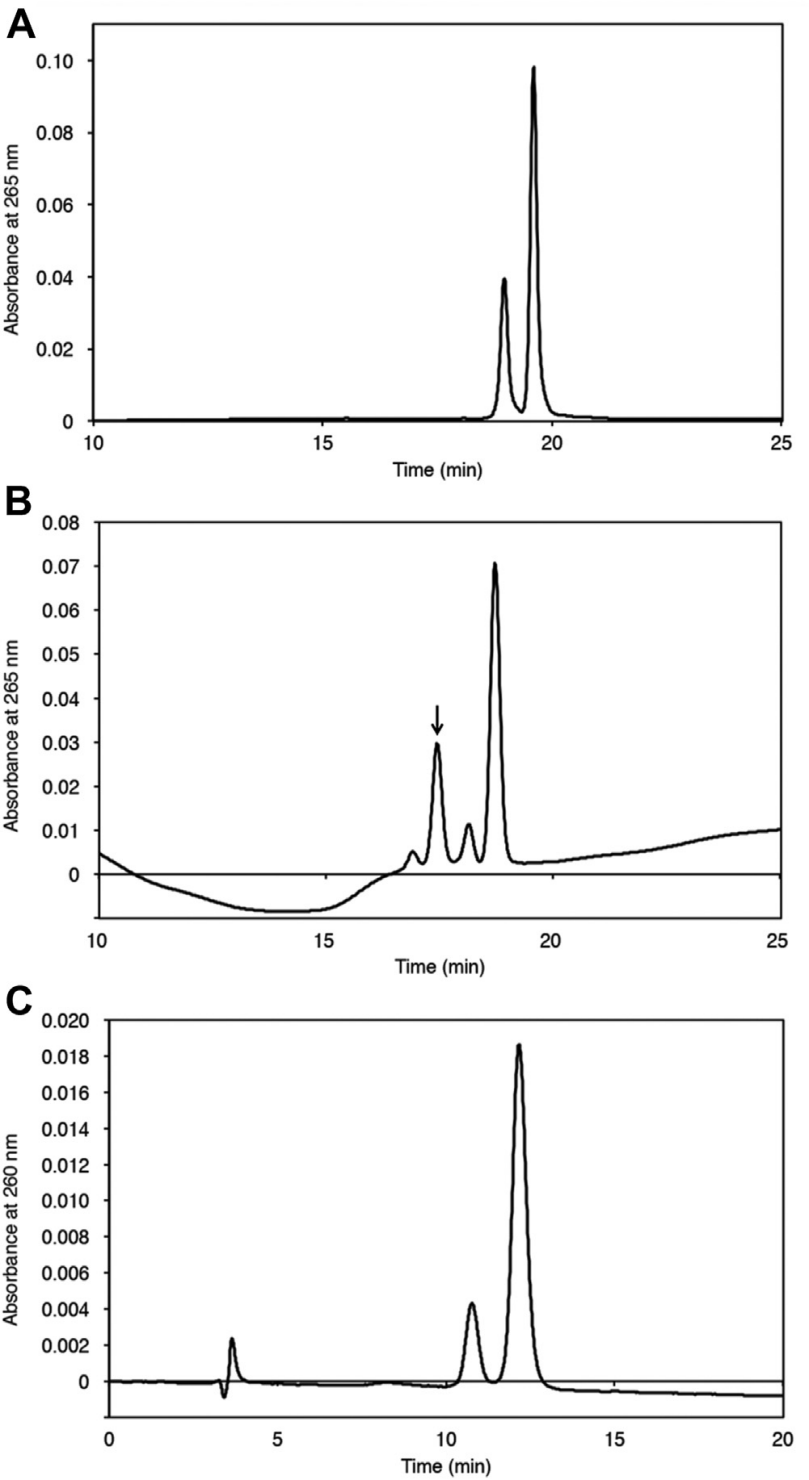
**Preparation of the oligonucleotide containing GA-FAPy-dfG.***A* and *B*, HPLC analysis of the reaction mixture of p-d(TTTTfGTTTT) treated with GA. *A*, an Inertsil ODS-3 5 μm column (4.6 × 250 mm; GL Sciences) was used at a flow rate of 1 ml/min with 6 to 16% CH_3_CN gradient in 0.1 M TEAA. *B*, an Inertsil Ph-3 5 μm column (4.6 × 250 mm; GL Sciences) was used at a flow rate of 1 ml/min with 9 to 14% CH_3_CN gradient in 0.1 M TEAA. The last peak was the starting 9-mer in both cases, and the product indicated by an *arrow* was isolated. *C*, anion-exchange HPLC analysis of the reaction mixture after incubation of the GA^7^dfG-containing 30-mer at 37 °C for 20 h in 0.1 M sodium phosphate (pH 8.0). The elution conditions are described in the Experimental procedures section. The retention time of the GA^7^dfG 30-mer was 10.8 min, and the new peak eluted out at 12.2 min was proved to be the hydrolyzed product, GA-FAPy-dfG, by mass spectrometry after purification. GA, glycidamide; GA-FAPy-dfG, *N*^6^-(2-deoxy-2-fluoro-d-arabinofuranosyl)-2,6-diamino-3,4-dihydro-4-oxo-5-[*N*-(2-carbamoyl-2-hydroxyethyl)formamido]pyrimidine; TEAA, triethylammonium acetate.

Oligonucleotides containing the lesion were required to investigate the mutation caused by the GA-FAPy product. We intended to obtain an oligonucleotide containing GA^7^dG first and then to hydrolyze it to the FAPy form. However, the preparation of an oligonucleotide containing GA^7^dG is challenging owing to the cleavage of the labile glycosidic bond, resulting in the abasic site and N7-GA-Gua. To avoid this issue, we adopted the 2′-F isostere approach, wherein 2-deoxy-2-fluoro-β-d-arabinofuranose is used as the sugar moiety to increase the stability of the glycosidic bond at the GA^7^dG (37). As dfG, the 2′-F isostered dG, is also resistant to enzymatic cleavage by glycosylases (44), it is reasonable to expect that the removal of the lesion by base excision repair would also be inhibited. The building block to incorporate dfG into oligonucleotides is commercially available. Using this modified nucleoside, a 5′-phosphorylated 9-mer containing dfG surrounded by dT, p-d(TTTTfGTTTT), was synthesized on a DNA synthesizer. Only thymine was used for this sequence because dA and cytidine reportedly react with GA (12, 45), whereas the adduct with thymidine is formed in significantly low amounts at pH 7.0 (45). This oligonucleotide was treated with GA at 60 °C for 4 h in 50 mM sodium phosphate (pH 7.0). When the reaction mixture was analyzed by reversed-phase HPLC using an octadecyl-silica column, a single product peak was detected at a retention time slightly shorter than that of the starting oligonucleotide (Fig. 3*A*). However, when a phenyl column was used, two small peaks were observed (Fig. 3*B*). The major product separated on the phenyl column was isolated, and MS analysis (Fig. S1*A*) revealed that this product contained the GA adduct. By ligating the 9-mer with the flanking sequences using a splint oligonucleotide (Fig. S1*B*), followed by HPLC purification, a 30-mer oligonucleotide containing the GA adduct at the modified nucleoside (GA^7^dfG) was obtained. A solution of this product in 0.1 M sodium phosphate (pH 8.0) was incubated at 37 °C for 20 h, and the mixture was analyzed by anion-exchange HPLC (Fig. 3*C*). The larger peak with a longer retention time that emerged after the incubation was isolated, and the mass spectrometric analysis (Fig. S1*C*) suggested that its molecular weight coincided with that of the 30-mer containing *N*^6^-(2-deoxy-2-fluoro-d-arabinofuranosyl)-2,6-diamino-3,4-dihydro-4-oxo-5-[*N*-(2-carbamoyl-2-hydroxyethyl)formamido]pyrimidine (GA-FAPy-dfG).

### GA-FAPy-dfG on the template strand severely inhibits Pol activity

**Figure 4:**
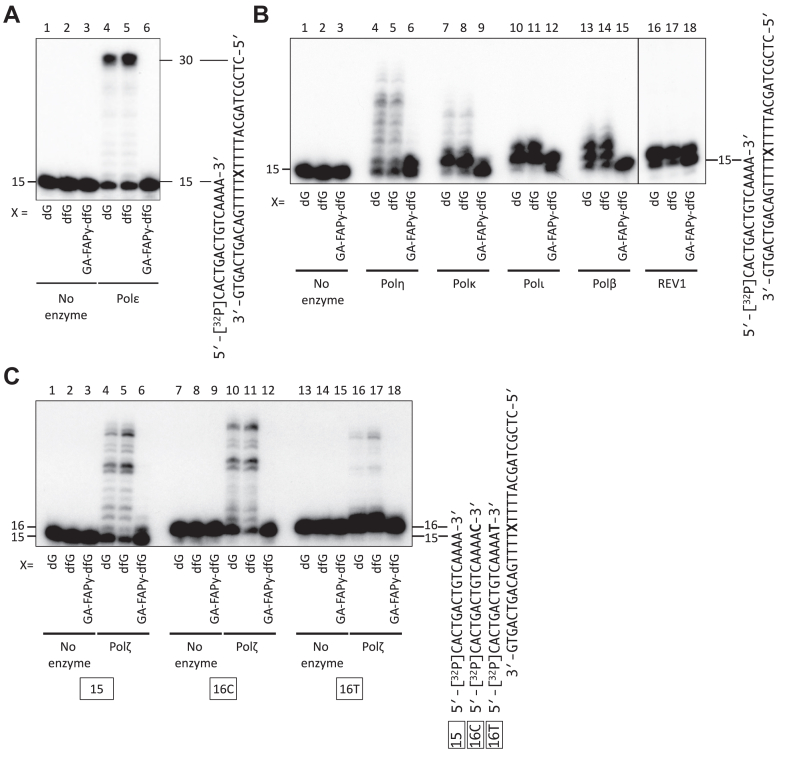
**Effects of GA-FAPy-dfG on DNA polymerases activity.***A*, primer extension assay past GA-FAPy-dfG by Polε. A [^32^P]-labeled 15-mer primer was annealed with 30-mer template oligonucleotides carrying dG (lanes 1 and 4), dfG (lanes 2 and 5), or GA-FAPy-dfG (lanes 3 and 6) at the 16th nucleotide from the 3′ terminus (denoted as X). The primer–template substrates were then incubated with (lanes 4–6) or without (lanes 1–3) Polε for 15 min at 37 °C. The products were subjected to polyacrylamide gel electrophoresis under denaturing conditions and then autoradiographed. *B*, primer extension assay past GA-FAPy-dfG by Polη, Polκ, Polι, Polβ, and REV1. A [^32^P]-labeled 15-mer primer was annealed with 30-mer template oligonucleotides carrying dG (lanes 1, 4, 7, 10, 13, and 16), dfG (lanes 2, 5, 8, 11, 14, and 17), or GA-FAPy-dfG (lanes 3, 6, 9, 12, 15, and 18) at the 16th nucleotide from the 3′ terminus (denoted as X). The primer–template substrates were then incubated with Polη (lanes 4–6), Polκ (lanes 7–9), Polι (lanes 10–12), Polβ (lanes 13–15), REV1 (lanes 16–18), or without enzymes (lanes 1–3) for 15 min at 37 °C. *C*, primer extension assay by Polζ using 16-mer primers, 16C and 16T, which placed the 3′ dC or dT of the primer opposite dG, dfG, or GA-FAPy-dfG. The [^32^P]-labeled primers (15, lanes 1–6; 16C, lanes 7–12; 16T, lanes 13–18) were annealed with 30-mer template oligonucleotides carrying dG (lanes 1, 4, 7, 10, 13, and 16), dfG (lanes 2, 5, 8, 11, 14, and 17), or GA-FAPy-dfG (lanes 3, 6, 9, 12, 15, and 18) at the 16th nucleotide from the 3′ terminus (denoted as X). The primer–template substrates were then incubated with (lanes 4–6, 10–12, and 16–18) or without (lanes 1–3, 7–9, and 13–15) Polζ for 15 min at 37 °C. The products were subjected to polyacrylamide gel electrophoresis under denaturing conditions and then autoradiographed. GA-FAPy-dfG, *N*^6^-(2-deoxy-2-fluoro-d-arabinofuranosyl)-2,6-diamino-3,4-dihydro-4-oxo-5-[*N*-(2-carbamoyl-2-hydroxyethyl)formamido]pyrimidine.

To test whether GA-FAPy-dfG on template DNA directly inhibits Pol activity, we performed primer extension assays using the 30-mer oligonucleotides carrying dG, dfG, or GA-FAPy-dfG, at the 16th nucleotide from the 3′ terminus of the template DNA. The catalytic subunit of human Polε extended a 15-mer primer on both the dG and dfG templates with similar efficiencies. In contrast, the primer extension by Polε was drastically inhibited by GA-FAPy-dfG (Fig. 4*A*). We then examined whether major TLS polymerases, Polη, Polκ, Polι, and REV1 (46), can bypass GA-FAPy-dfG. GA-FAPy-dfG on the template strand severely inhibited primer extension by all the tested Pols (Fig. 4*B*). Notably, they incorporated a single base opposite GA-FAPy-dfG; however, almost no further extended products were observed. We also examined whether Polβ, which has been reported to bypass N7-dG monoadducts (47), can incorporate nucleotides opposite GA-FAPy-dfG. In contrast to the Y-family Pols, no nucleotide incorporation was observed with Polβ.

It has been postulated that TLS is a multiple step DNA synthesis opposite a lesion (48). First, “inserter” Pols incorporate nucleotides into the nascent strand terminus at the opposite site of the lesion and then “extender” Pols extend the terminus further to complete TLS. Therefore, we examined whether the nucleotide incorporated opposite GA-FAPy-dfG could be further extended by Polζ, the major “extender,” with the ability to extend from mispaired primer terminus. To test this, we designed 16-mer primers, 16C and 16T, which had dC or dT at their 3′ terminus. The annealing of these primers to the 30-mer template placed the 3′ dC or dT of the primers opposite to dG, dfG, or GA-FAPy-dfG, which recapitulated the extension from incorporated nucleotides opposite the lesion. When the template had dG or dfG, Polζ could extend the primers more efficiently from both the 15-mer and paired 16-mer (16C) termini than from the mispaired 16-mer (16T) terminus (Fig. 4*C*). When the template had GA-FAPy-dfG, elongation from neither paired nor mispaired 16-mer termini opposite the lesion was observed, even though slight incorporations of one or two nucleotides from the 15-mer terminus were observed. These results indicate that GA-FAPy-dfG severely inhibited the extension of the primer terminus from incorporated nucleotides opposite the lesion, by Polζ.

### GA-FAPy-dfG inhibits DNA replication and induces point mutations in human cells

**Figure 5:**
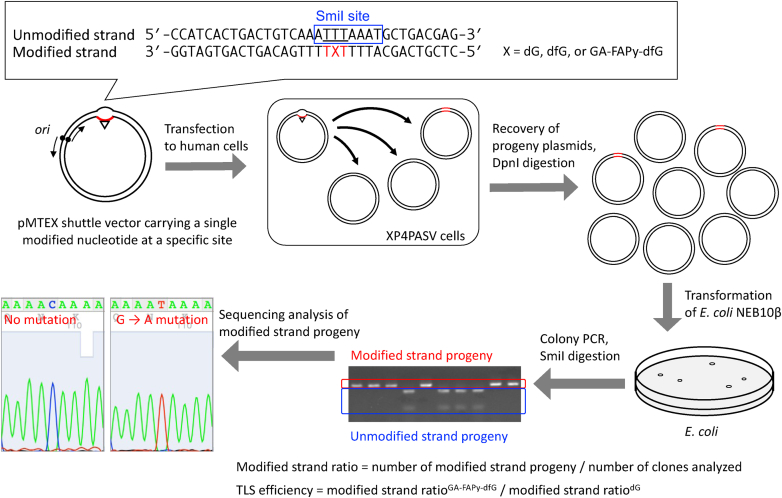
**Schematic diagram of the intracellular TLS assay using a shuttle vector.** dG, dfG, and GA-FAPy-dfG were introduced at a specific position (denoted as X) on one side of the shuttle vector (modified strand), whereas the other strand (unmodified strand) had a three-base mismatch (*underlined*) for the GA-FAPy-dfG and SmiI recognition sequence (*blue box*). The vector was transfected into XP4PASV cells and allowed to replicate for 48 h. Progeny plasmids were then recovered and digested with DpnI to remove nonreplicated original DNA. *Escherichia coli* was transformed with progeny plasmids, and resultant clones were subjected to PCR to amplify the lesion site. The PCR products were treated with SmiI to digest unmodified strand progeny. Replication efficiency was calculated from the ratio of the modified strand progeny in the recovered plasmids and is shown relative to dG. Mutation spectra were analyzed using Sanger sequencing of the PCR products of the modified strand progeny. GA-FAPy-dfG, *N*^6^-(2-deoxy-2-fluoro-d-arabinofuranosyl)-2,6-diamino-3,4-dihydro-4-oxo-5-[*N*-(2-carbamoyl-2-hydroxyethyl)formamido]pyrimidine; TLS, translesion DNA synthesis.

A schematic representation of the site-specific replicative TLS assay is shown in Figure 5. To examine whether GA-FAPy-dfG on the template strand affects DNA replication, the pMTEX-GA2 shuttle vector carrying dG, dfG, or GA-FAPy-dfG at the same position (Fig. S2, *A* and *B*) was transfected into XP4PASV cells, a simian virus 40 (SV40)–transformed human skin fibroblast cell line defective in xeroderma pigmentosum group C (XPC) protein. GA-FAPy-dfG is located on three consecutive mismatches to discern the progeny derived from unmodified or modified strands. As only unnicked covalently closed circular DNA (cccDNA) was transfected, it is unlikely that mismatch repair would remove the mismatched bases. However, global genome nucleotide excision repair can easily eliminate the lesion. The XPC complex, which detects DNA damage in global genome nucleotide excision repair, binds to sites with DNA helix distortions; therefore, even lesions with low affinity for XPC (*e.g.*, *cis-syn* cyclobutane pyrimidine dimers) can be efficiently removed if they are located at opposite mismatched bases (49). The use of XPC-deficient cells enables precise measurements of lesion bypass efficiency. Moreover, even if the lesions on the mismatched bases were unexpectedly removed from the cells, it would not affect the mutation spectrum of the modified strand because its sequence should be converted to that of the unmodified strand during the repair process, and therefore, excluded from mutation analysis.

**Figure 6:**
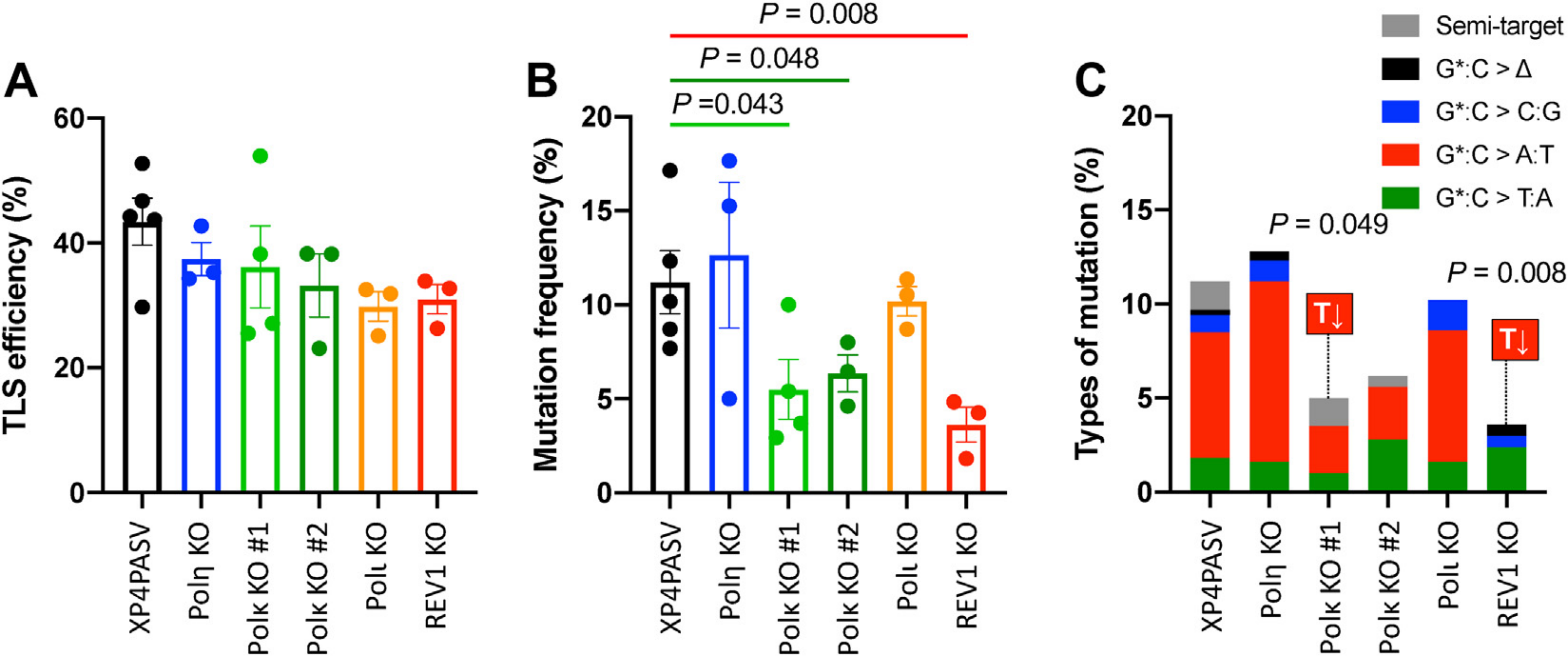
**Effects of polymerase deficiency on the bypass efficiency and fidelity of GA-FAPy-dfG.** TLS efficiency (*A*) and mutation frequency (*B*) of GA-FAPy-dfG in a series of isogenic TLS polymerase KO cells. *C*, mutation spectrum at the site of GA-FAPy-dfG. Assays were performed more than thrice, independently. GA-FAPy-dfG, *N*^6^-(2-deoxy-2-fluoro-d-arabinofuranosyl)-2,6-diamino-3,4-dihydro-4-oxo-5-[*N*-(2-carbamoyl-2-hydroxyethyl)formamido]pyrimidine.

As shown in Fig. S3*A*, the replication efficiency of the GA-FAPy-dfG strand in XP4PASV cells was significantly lower than that of both dG and dfG in comparison with that of the dG strand. This indicated that GA-FAPy-dfG strongly inhibited DNA replication. However, although replication efficiency of dfG was slightly lower than that of dG, there was no statistically significant difference, indicating that dfG on the template strand had little interference with DNA replication. As the “inserter” TLS Pols (*i.e.*, Polη, Polκ, Polι, and REV1) showed activity to incorporate at least one nucleotide opposite GA-FAPy-dfG, we generated a series of these “inserter” TLS Pol–deficient XP4PASV cells using CRISPR–Cas9 genome editing to examine which TLS Pol is responsible for the GA-FAPy-dfG–induced mutagenesis. The disruption of the target genes was confirmed by genomic sequencing (Fig. S4) and Western blotting (Fig. S5). These isogenic TLS Pol KO cells exhibited comparable replication efficiency of the GA-FAPy-dfG strand with their parental XP4PASV cells (Fig. 6*A*). On examining the mutation spectrum of the modified strand progeny, we found almost no mutations in either dG or dfG. In contrast, targeted mutations (mutations at the site of the lesion) were specifically observed in the GA-FAPy-dfG strand (Fig. S3*B*), in which the most frequent mutation was G∗:C > A:T transition (G∗ indicates the modified nucleotide), caused by incorporation of the incorrect nucleotide, dT, opposite GA-FAPy-dfG (6.7%), followed by G∗:C > T:A transversion (1.8%) (Table S1). The mutation frequency of GA-FAPy-dfG was significantly decreased in two independent clones of Polκ KO (5.51% and 6.36%) and REV1 KO cells (4.26%) compared with XP4PASV cells (11.21%) (Fig. 6*B*). Regarding the mutation spectra, the G∗:C > A:T transition was also dominant in Polη and Polι KO cells; however, significant decrease or decreasing tendencies of the G∗:C > A:T transition were observed in the two independent clones of Polκ KO cells (2.5% and 2.8%) compared with XP4PASV cells (6.7%) (Fig. 6*C* and Table S1). More prominently, the G∗:C > A:T transition was completely abolished in REV1 KO cells. In addition to the decrease in G∗:C > A:T transition, no G∗:C > C:G transversion was observed in Polκ KO cells, whereas only few clones exhibited this mutation in the parental XP4PASV cells (0.9%), with this difference not being statistically significant. Although a small number of semitargeted mutations (mutations within the three nucleotides next to the lesion) were observed in XP4PASV and Polκ KO cells, the difference was not statistically significant among the cells tested.

**Figure 7:**
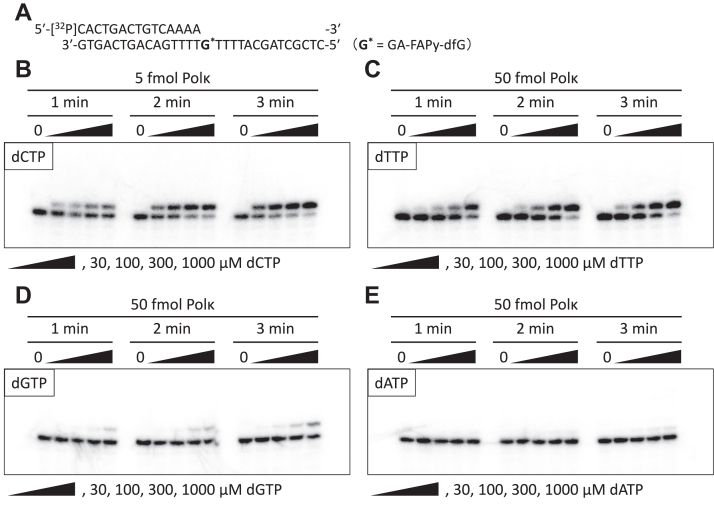
**Single nucleotide incorporation by Polκ.** A [^32^P]-labeled 15-mer primer annealed with 30-mer template oligonucleotides carrying GA-FAPy-dfG at the 16th nucleotide from the 3′ terminus (*A*) was incubated with 5 fmol (for dCTP [*B*]) or 50 fmol (for dTTP [*C*], dGTP [*D*], and dATP [*E*]) of Polκ in the presence of one of the indicated dNTPs (30–1000 μM) for indicated periods at 37 °C. The products were subjected to polyacrylamide gel electrophoresis under denaturing conditions and then autoradiographed. GA-FAPy-dfG, *N*^6^-(2-deoxy-2-fluoro-d-arabinofuranosyl)-2,6-diamino-3,4-dihydro-4-oxo-5-[*N*-(2-carbamoyl-2-hydroxyethyl)formamido]pyrimidine.

As REV1 is a unique deoxycytidyl transferase that specifically incorporates dCTP (50, 51, 52), we focused on Polκ as an error-prone inserter and examined whether it could incorporate incorrect nucleotides opposite GA-FAPy-dfG. Polκ predominantly incorporated correct dCTP opposite GA-FAPy-dfG, but it also weakly incorporated incorrect dTTP and slightly incorporated dGTP (Fig. 7). However, incorporation of dATP was hardly observed. As the misincorporation potential of dTTP was consistent with the mutation spectrum of the intracellular TLS assay, we examined the misincorporation frequency (*f*_ins_) of dTTP opposite GA-FAPy-dfG by Polκ. Steady-state kinetic analyses revealed that dTTP exhibits lower affinity (higher *K*_*m*_ value) than dCTP (Fig. S6). The *f*_ins_, which represents the ratio of the catalytic efficiency (*k*_cat_/*K*_*m*_) of dTTP incorporation compared with dCTP, was 4.12 × 10^−2^ (Table 1).

**Table 1:** Steady-state kinetics of dCTP and dTTP incorporation opposite GA-FAPy-dfG by Polκ

dNTP	*K*_*m*_ (μM)	*k*_cat_ (min^−1^)	*k*_cat_/*K*_*m*_ (μM^−1^ min^−1^)	*f*_ins_^a^
dCTP	226.6	110.6	0.488	—
dTTP	1054	21.18	0.020	4.12

a *f*_ins_ = (*k*_cat_/*K*_*m*_)_dTTP_/(*k*_cat_/*K*_*m*_)_dCTP_.

### Molecular modeling of base pairing with GA-FAPy-dG

**Figure 8:**
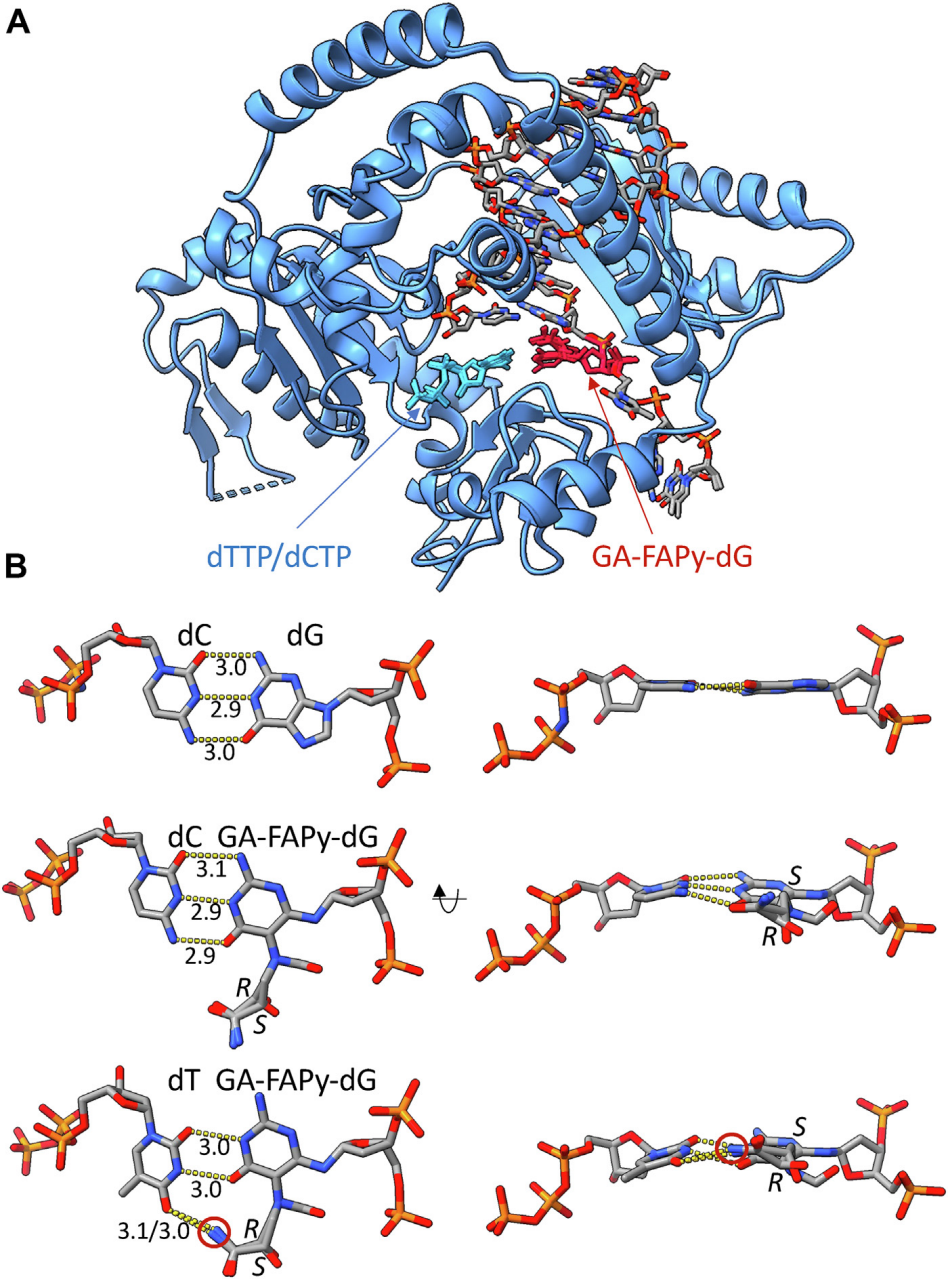
**Molecular modeling for the base pairings of dC and dT with GA-FAPy-dG.***A*, superimposed models of human Polκ in complex with dNTP and dsDNA containing GA-FAPy-dG. The protein and DNA/nucleotide are shown in *blue ribbon* models and element-colored stick models, respectively. The incorporating dTTP/dCTP and base pairing GA-FAPy-dG residue are colored *light blue* and *red*, respectively. *B*, *top* (*left*) and *side* (*right*) close-up views of the GA-FAPy-dG base pairs. The presented models are canonical dC:dG (*top*), dC:GA-FAPy-dG (*middle*), and dT:GA-FAPy-dG (*bottom*). The H-bonds between bases are indicated with *dotted yellow lines*, and the bonding distances (Å) are noted. *R*- and *S*-isomers were superposed for GA-FAPy-dG models and indicated at the chiral centers. GA-FAPy-dfG, *N*^6^-(2-deoxy-2-fluoro-d-arabinofuranosyl)-2,6-diamino-3,4-dihydro-4-oxo-5-[*N*-(2-carbamoyl-2-hydroxyethyl)formamido]pyrimidine.

The conformations of GA-FAPy base pairs have not yet been experimentally revealed. Therefore, a possible conformation was examined to evaluate whether GA-FAPy could form a reasonable base pair with dC or dT in the active site of Pol by constructing structural models. The structural model of GA-FAPy-dG was built into the crystal structure of human Polκ (53) opposite to the incorporating dCTP or dTTP (Fig. 8*A*). Epimerization occurs at the anomeric carbon in alkyl-FAPy-dG (26). Another issue in stereochemistry is the carbon atom bearing a hydroxyl group in the N7-GA adduct. The GA-FAPy-dGs in the cells are assumed to be a mixture of stereoisomers, mainly α or β anomer at C1 of deoxyribose and nontautomeric *R* or *S* isomer at the carbon atom {CH_2_C∗(H)(OH)CO(NH_2_)} in the GA residue. Under α anomer configuration, the GA-FAPy-dG base pointed away from the opposite DNA strand, and no reasonable conformation was conceived for base pairing. Moreover, purine bases, A or G, were not properly modeled, because no space was available for them at the active site to form a base pair with GA-FAPy-dG. Therefore, the GA-FAPy-dG in β-anomer was modeled for two nontautomeric stereoisomers *R* and *S* and base paired with dC or dT (Supporting Information S1–S4). After annealing the structural models, the base pairing nucleotides were extracted and the potential energies were evaluated with MMFF94x (54) and Amber14EHT (55) forcefields (Table S2).

The canonical dC:dG pair was stable with less than −660 kcal/mol potential energy. The GA-FAPy-dG base pairs were rather unstable with −300 to 350 kcal/mol, most probably owing to the loss of base pair planarity, caused by the insertion of the amide group between the pyrimidine ring and deoxyribose (Fig. 8*B*). dC was formerly known to be incorporated against FAPy-dG, since three H-bonds of dC:dG can be maintained, although in a distorted geometry, for base pairing (56). For dT, the model suggested two H-bonds between the thymine and guanine base residue, and in addition, one H-bond between the carbonyl (O4) of thymine base and the amino group of the GA residue (*red circled* in Fig. 8*B*) was potentially formed. This H-bond seemed to be maintained in both *R*- and *S*-isomers and likely provided additional stability to the dT:GA-FAPy-dG base pair. As a result, the potential energies were roughly comparable between dC:GA-FAPy-dG and dT:GA-FAPy-dG base pairs. This trend was also consistent for the electrostatic potential components, which included the H-bonding energies (Table S2). Thus, the models suggested that dT might be selected along with dC against the GA-FAPy-dG nucleotide in the template DNA.

## Discussion

In this study, we focused on the mutagenic potential of the FAPy-type product of GA-adducted guanine. The formation of GA-FAPy-dG was confirmed at the nucleoside level by the treatment of dG with GA in a pH 7.0 buffer at 37 °C, followed by HPLC and LC–MS analyses (Fig. 2). Based on an estimated molecular ratio of AP sites to GA-FAPy-dG of 16.5:1, it was hypothesized that approximately 5.7% of GA^7^dG was converted to GA-FAPy-dG through ring-opening hydrolysis. Although it remains unknown whether the second molecule of GA reacts with GA^7^dG before or after glycoside-bond cleavage, the second adduct formation would facilitate the glycoside-bond cleavage by the inductive effect of the hydroxyl group. Hence, even in the first scenario, it is likely that the dG adducted with two GA molecules would not be retained in the genomic DNA.

The problem in the preparation of DNA containing this damaged base was the lability of the glycosidic bond of the precursor GA^7^dG. Since the glycosidic-bond cleavage would reduce the yield of the desired product to a large extent, we utilized 2′-F isostere that stabilizes N7-dG adducts (37). Indeed, apurinic products were not detected in the electrospray ionization–MS analysis of both 9- and 30-mer oligonucleotides containing GA^7^dfG. It has been reported that FAPy derivatives were predominantly produced from the N7-dG adduct under strongly basic conditions (57, 58). In contrast, the GA-FAPy product was detected in the mixture after the GA treatment of dG in a pH 7.0 buffer at 37 °C in our study, suggesting that GA-FAPy-dG is produced under physiological conditions. Unlike N7-dG adducts, FAPy is chemically stable; therefore, GA-FAPy-dG formation upon exposure to GA has not been detected *in vivo* by conventionally used neutral thermal hydrolysis. However, after a single administration of methylating agents (*e.g.*, *N*-*N*-dimethylnitrosamine and 1,2-dimethylhydrazine) to rats, it has been reported that in contrast to N7-Me-dG, time-course accumulation of Me-FAPy-dG was observed in liver genomic DNA (59), suggesting that alkylated FAPy, which is chemically more stable, accumulates in the genome. As FAPy derivatives are considered to be more mutagenic than their parental N7-dG adducts, GA-FAPy-dG was suspected to be responsible for the acrylamide-induced mutagenesis. Our study revealed that the DNA replication efficiency of the modified strand carrying GA-FAPy-dfG was significantly lower than that of dG and dfG, indicating that GA-FAPy-dG on genomic DNA could be a major obstacle to DNA replication. Our data indicated that GA-FAPy-dfG directly inhibited primer extension by high-fidelity replicative DNA Polε, which is responsible for leading strand DNA synthesis. Moreover, GA-FAPy-dfG severely inhibited DNA synthesis not only by DNA Polε but also by a series of TLS polymerases with the ability to bypass various DNA lesions (29). FAPy derivatives are known to interconvert between α and β anomers, and the α anomers of FAPy-dG and AFB_1_-FAPy-dG block DNA replication (58, 60, 61). Although the anomerization potential of GA-FAPy-dG in ssDNA and dsDNA remains elusive, GA-FAPy-α-dG may participate in the inhibition of DNA replication. Notably, most of these DNA Pols (except Polβ) could incorporate a single nucleotide opposite GA-FAPy-dfG, suggesting that GA-FAPy-dfG may be bypassed by a two-step TLS model. In the model, the first Pol (inserter) inserts a single or few nucleotides opposite a lesion, and the second Pol (extender) subsequently extends the daughter strand further to complete the bypass of the lesion (48). However, the Pols responsible for the second step in the bypass of GA-FAPy-dfG remain unclear. No further elongation was observed from the nucleotide incorporated opposite GA-FAPy-dfG by Polζ, which is a possible candidate for extender TLS Pol (62). The severe inhibition of DNA replication by GA-FAPy-dG was consistent with the fact that acrylamide and its metabolite GA are clastogenic and induce chromosomal aberrations and sister chromatid exchanges (63, 64) because the inhibition of DNA replication and subsequent replication fork collapse lead to chromosomal instability.

The mutation frequency of GA-FAPy-dfG was significantly higher than that of dG and dfG, indicating that GA-FAPy-dG is a promutagenic DNA lesion similar to other FAPy derivatives (19, 20, 21, 22, 23, 24, 25, 26). GA-FAPy-dfG induced over 10% of point mutations at the lesion, which was much higher than that induced by the *cis-syn* cyclobutene–pyrimidine dimer, the most common ultraviolet-induced lesion (65). Thus, spontaneous hydrolysis of GA^7^dG can cause DNA replication inhibition and subsequent TLS using error-prone TLS polymerases, resulting in induction of mutations at the lesion.

The mutation spectrum analysis revealed that GA-FAPy-dfG specifically induced targeted mutations at the lesion, suggesting that GA-FAPy-dG–induced base substitutions are caused at the “insertion” step of TLS. The primer extension assay showed that none of the individual Pols tested, including Polε, Polη, Polκ, Polι, Polβ, REV1, and Polζ, could bypass GA-FAPy-dfG solely through their own activity. However, several TLS Pols (*i.e.*, Polη, Polι, Polκ, and REV1) could incorporate at least a single nucleotide opposite GA-FAPy-dfG, suggesting that these Pols may contribute to GA-induced single-base substitutions. Indeed, there was no difference in intracellular TLS efficiency among a series of “inserter” polymerase KO cells, suggesting redundancy in the bypass activity of GA-FAPy-dfG as well as (6–4) pyrimidine–pyrimidone photoproducts (65). On the other hand, the mutation frequency was decreased in Polκ KO cells and REV1 KO cells, indicating that these TLS polymerases contribute to error-prone TLS past GA-FAPy-dfG. Mutation spectrum analysis revealed that Polκ and REV1 contributed to the most prominent G∗:C > A:T transition (*i.e.*, they mainly contributed to dT incorporation opposite GA-FAPy-dfG). The consistency between the reduced frequency of G∗:C > A:T transition in Polκ KO cells compared with XP4PASV cells (6.7% in XP4PASV *versus* 2.5% in Polκ KO #1 and 2.8% in Polκ KO #2) and the catalytic activity of Polκ (*f*_ins_ of dTTP opposite GA-FAPy-dfG, 4.12 × 10^−2^) suggests that the catalytic activity of Polκ directly contributes to the G:C > A:T transition by incorporating the incorrect dTTP opposite GA-FAPy-dfG. The fact that G∗:C > A:T transition was still observed even in Polκ KO cells indicates a redundancy in the dT incorporation opposite GA-FAPy-dfG by other TLS Pols. However, comparable decreases in the G∗:C > A:T transition in two independent clones of Polκ KO cells strongly suggest that it is Polκ that mainly contributes to the dT incorporation opposite GA-FAPy-dfG.

More strikingly, the G∗:C > A:T transition was completely abolished in REV1 KO cells. It is noteworthy that REV1 has characteristic deoxycytidyl transferase activity using its own protein template (66), which solely incorporates dC regardless of the template DNA sequence. Thus, when REV1 incorporates nucleotides opposite GA-FAPy-dfG, the correct dC should be incorporated. Besides the dCMP transferase activity, REV1 has another key role as a scaffold for polymerase switching through interactions with other TLS polymerases *via* its C-terminal region (67, 68, 69, 70). Therefore, we hypothesized that Polκ, which has dTTP incorporating activity opposite GA-FAPy-dfG, incorporated a single nucleotide opposite GA-FAPy-dG and then switched to the “extender” polymerase *via* the interaction with REV1. Although a homoallelic disruption of *REV3*, which encodes the catalytic subunit of Polζ in cells, was not tested owing to cellular lethality in XP4PASV cells, the primer extension assay indicates that Polζ does not have an extension activity from either the paired or the mispaired terminus opposite GA-FAPy-dfG, suggesting that an unidentified Pol participates in the bypass of GA-FAPy-dG as an alternative extender.

In addition to the G∗:C > A:T transition, GA-FAPy-dfG is also responsible for the occurrence of all other types of mutations observed in the present assay, namely G∗:C > C:G and G∗:C > T:A transversions. Although there was no statistically significant difference in the frequency of G∗:C > C:G between XP4PASV and Polκ KO cells, the absence of this mutation in Polκ KO cells suggests that Polκ may also contribute to dG incorporation. Conversely, the frequency of G∗:C > T:A was comparable in all TLS Pols KO cells tested, implying the involvement of other Pols in this type of mutation through the incorporation of dA opposite GA-FAPy-dfG.

Despite the major conformational change of the guanosine ring opening, dC was still the most frequently incorporated base opposite to GA-FAPy-dfG. This was consistent with other FAPy derivatives, whose mutagenicity in mammalian cells has been reported (20, 21, 22, 23), except for the significantly bulky AFB_1_-FAPy-dG (24). Molecular modeling shows that the remaining pyrimidine ring of GA-FAPy-dG can maintain H-bonds similar to those of normal dC:dG pairs, implying that GA-FAPy-dG can form base pairs with dC. On the other hand, the loss of base pair planarity in the dC:FAPy-dG pair explains the mutagenic potential of GA-FAPy-dG. Although the major mutation induced by other FAPy derivatives, such as FAPy-dG (20, 21, 22), Me-FAPy-dG (23), and AFB_1_-FAPy-dG (24), was G:C > T:A transversion in mammalian cells, GA-FAPy-dfG showed a distinctive mutation spectrum, inducing mainly G:C > A:T transitions. The molecular model of dT:GA-FAPy-dG base pairing in the catalytic center of Polκ explained that 2-carbamoyl-2-hydroxyethyl group at *N*^5^ position of GA-FAPy-dG can form an additional H-bond with dT under β configuration, thereby contributing to the characteristic mutation spectrum of GA-FAPy-dG. The discrepancy between G∗:C > A:T transition was observed to be less than 10% in transfected XP4PASV cells, even though the potential energies were comparable between dC:GA-FAPy-dG and dT:GA-FAPy-dG base pairs and may be explained by the lower catalytic activity for the incorporation of dTTP compared with dCTP opposite GA-FAPy-dG, by Polκ.

Previous reporter gene mutation assays have reported that GA exposure significantly increases C:G > A:T transversions in the *cII* transgene in big blue (BB) mouse embryonic fibroblasts (30). As GA^7^dG is prone to depurination, it has been considered that the “A rule,” wherein A is preferentially incorporated opposite AP sites (71), may promote this type of mutation. However, the *cII* transgene mutation spectra of GA in animal organs varied: G:C > T:A and −1/+1 frameshifts in a homopolymeric run of Gs in the liver (31), A:T > G:C transition, and G:C > C:G transversion in the testes (33); A:T > T:A and G:C > C:G transversions and −1/+1 frameshifts at a homopolymeric run of Gs in the lungs (13); G:C > T:A, A:T > T:A, and A:T > C:G transversions in the brain (34) of BB mice; and no statistically significant differences were found in the thyroids of BB rats (32). The discrepancies between these experiments may be due to differences in animal species and organs, and mutagenesis assays using single reporter genes inherently have limitations in analyzing mutation spectra; that is, they only detect mutations in a limited region of the reporter genes, and only mutations that cause loss of functions of reporter genes can be detected. Thus, there is no consensus on the mutation spectrum of GA using a reporter gene mutation assay. Recent WES and WGS mutation assays have revealed that GA causes a broad spectrum of mutations with major single-base substitutions (SBSs) at both T:A and C:G base pairs. Mutations in the C:G, C > A mutation in trinucleotide contexts of 5′-C[C > A]A-3′, 5′-C[C > A]T-3′, and C > G mutation at 5′-G[C > G]C-3′ were enriched in the WES assay (35). In the WGS assay, all types of SBSs (*i.e.*, C > A, C > G, and C > T) were found in diverse sequence contexts except in triplets carrying G at their 3′-side base (5′-N[C > N]G-3′) (36). Notably, C > T transition and C > G transversion were also observed in these studies, besides the C > A transversion in SBSs at 5′-A[C > N]A-3′, which is the same trinucleotide context as in the complementary strand of GA-FAPy-dfG substrate used in these studies, consistent with the observation that GA-FAP-dfG induces C > T single-base substitution followed by C > A and C > G. Thus, our site-specific mutagenesis assay examined only one specific sequence context (5′-T[GA-FAPy-dfG]T-3′) owing to the limitation of substrate synthesis; however, the results were consistent with those obtained from the GA-induced mutation analyses using WES and WGS, indicating that the GA-FAPy-dfG-induced mutations observed in this study were consistent with the mutation signature of GA. Although the WES and WGS mutagenesis analyses could not distinguish whether mutations were derived from GA^7^dG, GA-FAPy-dG, or AP sites, our intracellular TLS assay using GA-FAPy-dfG demonstrates that GA-FAPy-dG could induce SBSs at the lesion site. Thus, GA-induced mutations at C:G may comprise mutations derived from mutagenic lesion bypasses at both AP sites and by GA-FAPy-dG, which mainly induce C > A and C > T, respectively.

In conclusion, our results indicate that GA^7^dG, a major acrylamide-induced DNA lesion, undergoes imidazolium ring–opening hydrolysis besides depurination under physiological conditions. The resulting GA-FAPy-dG inhibits DNA replication and induces point mutations, mainly G:C > A:T transition, as well as other base substitutions, at the lesion site. Thus, the FAPy derivative likely contributes to the genotoxicity and mutagenicity of GA. These findings provide novel insights into the mechanisms of acrylamide-induced mutagenesis and may therefore be useful for elucidating the effect of continuous mutation loads from dietary mutagen exposure.

## Experimental procedures

### Reaction of dG with GA

We added 50 μl of 1 M GA to a 50 μl solution of dG (0.10 μmol) in 0.2 M sodium phosphate (pH 7.0). After incubation at 37 °C for 48 h, an aliquot of this mixture was analyzed by reversed-phase HPLC using an Inertsil ODS-3 5 μm (4.6 × 250 mm; GL Sciences) with a linear gradient of acetonitrile (from 0 to 15%) in 0.1 M triethylammonium acetate (TEAA; pH 7.0), at a flow rate of 1.0 ml/min for 20 min. The data were acquired by a Waters 2998 photodiode array detector and processed using the Empower 3 software (Waters). For LC–MS analysis, the products (peaks i and ii in Fig. 2*A*) were isolated by monitoring absorption at 260 nm under the same HPLC conditions using ammonium acetate instead of TEAA, which was later removed by coevaporation with water.

### LC–MS analysis

LC–MS analysis was performed on a quadruple time-of-flight mass spectrometer (X500R quadruple time-of-flight; Sciex) coupled with a UPLC system (ACQUITY UPLC H-class plus; Waters). The peak i sample dissolved in water was injected onto the reversed-phase chromatographic column (InertSustain AQ-C18, 2.1 × 100 mm, 1.9 μm; GL Sciences) at a flow rate of 0.2 ml/min with a gradient of 0 to 40% of acetonitrile/water (95:5) for 7 min in 10 mM ammonium acetate. Hydrophilic chromatographic column (InertSustain Amide, 2.1 × 100 mm, 1.9 μm; GL Sciences) was used for peak ii sample at a flow rate of 0.25 ml/min with a gradient of 75 to 50% of acetonitrile/water (9:1) for 3 min in 0.1% (v/v) formic acid and 10 mM ammonium formate to improve separation between hydrophilic compounds. The mass spectrometer was operated in positive electrospray ionization mode with a capillary voltage of 5500 V. The source temperature was 350 °C, and declustering potential was 40 V. MS/MS measurement by collision-induced dissociation was performed to confirm the identity of each isolated sample. The precursor ions for MS/MS measurements were set to *m/z* 239.1 ([M + H]^+^ of N7-GA-Gua) and *m/z* 373.1 ([M + H]^+^ of GA-FAPy-dG). Collision energy for the two were 25 and 20 V, respectively. LC–MS grade acetonitrile and formic acid, ultrapure water, 1 mol/l ammonium acetate, and ammonium formate solutions for HPLC were obtained from FUJIFILM Wako Pure Chemical.

### Preparation of 30-mer oligonucleotide containing 2′-fluorine substituted analog of GA-FAPy-dG

A 5′-phosphorylated 9-mer containing dfG, p-d(TTTTfGTTTT), was synthesized using 2′-F-G-ANA-CE Phosphoramidite (Glen Research). An aliquot of the 9-mer (5 nmol) was treated with GA (5 μmol) in 50 mM sodium phosphate (pH 7.0, 50 μl) at 60 °C for 4 h. The major product was isolated under the HPLC conditions described in the legend to Figure 3*B*, and adduct formation was confirmed by MS (Fig. S1*A*). This procedure was repeated to obtain a sufficient amount of the product. The 9-mer, p-d(TTTTXTTTT), where X is GA^7^dfG (7.0 nmol), was mixed with d(CTCGTCAGCA) (10.5 nmol), p-d(GACAGTCAGTG) (10.5 nmol), and d(CTGTCAAAACAAAATGCTG) (8.4 nmol) (Fig. S1*B*) in water (53 μl). It was then mixed with a buffer containing 0.66 M Tris–HCl (pH 7.6), 66 mM MgCl_2_, 0.1 M DTT, and 1 mM ATP (to 7 μl). T4 DNA ligase (10 μl, 3500 units) was added. After incubation at 16 °C for 18 h, the mixture was analyzed by HPLC using a μBondasphere C18 5 μm 300 Å column (3.9 × 150 mm; Waters) at a flow rate of 1.0 ml/min with a 7 to 13% CH_3_CN gradient (20 min) in 0.1 M TEAA in a column oven set at 50 °C. The peak that emerged later than that of the splint 19-mer was isolated, and the eluate was desalted using an NAP-10 column (Cytiva). The 30-mer without the GA adduct was also prepared using p-d(TTTTfGTTTT) in the same manner. The GA^7^dfG-containing 30-mer was converted to the GA-FAPy-dfG-containing oligonucleotide by incubating it at 37 °C for 20 h in 0.1 M sodium phosphate (pH 8.0). The product was purified by anion-exchange HPLC using a TSK-GEL DEAE-2SW column (4.6 × 250 mm; Tosoh Corporation) at a flow rate of 1.0 ml/min with a 0.6 to 0.8 M HCOONH_4_ gradient for 20 min in 20% aqueous CH_3_CN (Fig. 3*C*). After concentrating the solution on a rotary evaporator, the eluate was desalted using an NAP-10 column, and the final product was analyzed by electrospray ionization–MS (Fig. S1*C*).

### Enzymes

Recombinant proteins of human DNA Polε catalytic fragment (exoproficient), Polι, Polβ, REV1, and yeast Polζ (Rev3/Rev7) were purchased from Enzymax. A recombinant human DNA Polκ catalytic fragment (1–560) with a 6× His tag at its C terminus was purchased from Bio Academia. A recombinant human DNA Polη catalytic fragment (1–511) with a 6× His tag at its C terminus was expressed in *Escherichia coli* Rosetta (DE3) and purified using sequential column chromatography on HiTrap DEAE Fast Flow, Ni–NTA agarose, and MonoS columns (GE Healthcare) as previously described (72).

### Primer extension assay

The 5′-[^32^P] primer–template DNA was prepared by mixing the 15- or 16-mer primers, which were labeled at the 5′ end using T4 polynucleotide kinase and [γ-^32^P] ATP; the 30-mer template DNA contained the lesion at a molar ratio of 1:2.5. Proteins were added to the final 10 μl of the reaction mixture containing 40 mM Tris–HCl (pH 7.5), 10 mM MgCl_2_, 100 μM each of the four dNTPs, 10 mM dithiothreitol, 250 μg/ml bovine serum albumin, and 1.6 nM 5′-[^32^P] primer–template DNA, and incubated at 37 °C for 15 min. The reaction was terminated by adding 10 μl of 98% formamide/10 mM EDTA, followed by heat denaturation. The products were electrophoresed on a 20% polyacrylamide/8 M urea gel and autoradiographed.

### Preparation of the GA-FAPy-dfG–containing shuttle vector for the intracellular TLS assay

The multiple cloning sequence of the pMTEX shuttle vector used for carrying ultraviolet-induced photolesions (65) was modified using the KOD-Plus-Mutagenesis Kit (TOYOBO) to obtain pMTEX-GA1, which has a complementary sequence to the 30-mer oligonucleotide carrying GA-FAPy-dfG, with a three-base mismatch opposite the lesion site (Fig. S2*A*). To construct a shuttle vector that can be replicated in human cells, the polyoma virus origin and polyoma T antigen of the pMETX-GA1 vector were removed by NdeI and ClaI digestion, and the chemically synthesized SV40 origin was ligated (Fig. S2*B*). The resulting vector, pMTEX-GA2, was replicated in SV40-transformed human cell lines. Heteroduplex shuttle vectors containing dG, dfG, or GA-FAPy-dfG on the modified strand were prepared as previously described (65). In brief, phosphorylated 30-mer oligonucleotides carrying dG, dfG, or GA-FAPy-dfG at a specific position were annealed to the single-stranded pMTEX-GA2 vector. The partially double-stranded DNA was treated with T4 Pol (TaKaRa Bio) and T4 DNA ligase (New England Biolabs) with dNTPs in NEBuffer 2 (New England Biolabs) to obtain cccDNA. The resulting cccDNA was purified using CsCl–ethidium bromide density gradient centrifugation, followed by dialysis against Tris–EDTA buffer (pH 8.0).

### Cell lines and culture

XP4PASV, an SV40-transformed human skin fibroblast cell line from an XPC patient, was cultured in Dulbecco’s modified Eagle’s medium (Nissui Pharmaceutical) supplemented with 10% fetal bovine serum, at 37 °C and 5% CO_2_.

### Genome editing

Guide RNAs targeting exon 3 of *POLH* (5′-TGGAGTCACTAGAAGTATGT-3′), exon 3 of *POLK* (5′-TACCATAGTGCACATTGACA-3′), exon 3 of *POLI* (5′-CAGTTGGTATTAGTTAATGG-3′), and exon 5 of *REV1* (5′-ACATTCCATATCAGCTGTAC-3′) genes were prepared by *in vitro* transcription using Precision gRNA Synthesis Kit (Thermo Fisher Scientific). *In vitro* transcribed guide RNAs amounting to 125 ng were transfected with 0.5 μg of TrueCut Cas9 v2 Nuclease (Thermo Fisher Scientific) into XP4PASV cells using Lipofectamine CRISPRMAX Cas9 Transfection Reagent (Thermo Fisher Scientific). After culturing for 48 h, cells were detached using TrypLE Express (Thermo Fisher Scientific), and single clones were isolated by limiting dilution. The clones were amplified in a 6 cm^2^ dish, and an aliquot was subjected to sequencing analysis. Clones harboring homoallelic disruption of the target genes by nonsense frameshift mutations were stored using Bambanker Direct (GC Lymphotec).

### Intracellular TLS assay

Cells (2.5 × 10^5^) were seeded in a 25 cm^2^ flask and cultured overnight before transfection. They were transfected with 0.5 μg of the substrate plasmids carrying dG, dfG, or GA-FAPy-dfG at a specific position using FuGENE HD transfection reagent (Promega). The cells were cultured for 48 h and detached using TrypLE Express. Progeny plasmids were recovered from the cells using the method described by Hirt (73). The recovered plasmids were treated with DpnI at 37 °C for 2 h to remove nonreplicated input DNA. NEB10β electrocompetent *E. coli* (*araD*139 Δ(*ara-leu*)7697 *fhuA lacX74 galK* (φ80 Δ(*lacZ*)*M15*) *mcrA galU recA1 endA1 nupG rpsL* (Str^r^) Δ [*mrr-hsdRMS-mcrBC*]; New England Biolabs) was transformed with the progeny plasmids and plated on 1× YT plates containing 100 μg/ml carbenicillin (Sigma–Aldrich) and 50 μg/ml blasticidin S (Kaken Pharmaceutical). To amplify the lesion site, the Carb^r^/BlaS^r^ clones (96 clones for dG and dfG strands and 192 clones for GA-FAPy-dfG strand in each experiment) were picked up, suspended in 20 μl of SuperFi II DNA Polymerase (Thermo Fisher Scientific) reaction mixture (Platinum SuperFi II DNA Polymerase, 1× SuperFi II Buffer, 125 μM dNTPs, 100 nM forward/reverse primers), and subjected to PCR using the following primers: d(GTGCTTCTCGATCTGCATCCTG) (forward) and d(TGCCACTCATCGCAGTCGAGCT) (reverse). The amplification conditions were as follows: initial denaturing phase at 98 °C for 5 min; 35 cycles at 94 °C for 30 s, 60 °C for 30 s, and 72 °C for 30 s; and a final extension at 72 °C for 7 min. The PCR products were treated with FastDigest SmiI (Thermo Fisher Scientific) at 37 °C for 30 min to digest the unmodified strand progeny. To analyze the mutation spectra, the PCR products of the modified strand progeny were subjected to sequencing analysis using the pMTEX-seq primer: d(ATTCGTGAATTGCTGCCCTC). The replication efficiency of the modified strand was calculated using the number of modified strand progeny divided by the number of sequences analyzed; the dG strand in each experiment was set to 100%. The mutation frequency was calculated as the number of mutants detected divided by the number of clones analyzed. Each experiment was triplicated independently for each clone.

### Single nucleotide incorporation and steady-state kinetics assays

The 5′-[^32^P] primer–template DNA was prepared by mixing the 15-mer primers, which were labeled at the 5′ end using T4 polynucleotide kinase and [γ-^32^P] ATP, with the 30-mer template DNA containing the lesion at a molar ratio of 1:1.25. Polκ (5 or 50 fmol) was added to the final 10 μl of the reaction mixture containing 40 mM Tris–HCl (pH 7.5), 10 mM MgCl_2_, 30 to 1000 μM of respective dNTPs, 10 mM dithiothreitol, 250 μg/ml bovine serum albumin, and 100 nM 5′-[^32^P] primer–template DNA and incubated at 37 °C. A 2 μl aliquot of each sample was collected every minute for a total of 3 min. For steady-state kinetic assays, 2 fmol Polκ and 30 to 1000 μM dCTP or 10 fmol Polκ and 100 to 3000 μM dTTP were used. The reaction was terminated by adding an equal volume of 98% formamide/10 mM EDTA to the aliquot, followed by heat denaturation. The products were electrophoresed on a 20% polyacrylamide/8 M urea gel and autoradiographed. The steady-state velocities (*V*_0_) of dNTP incorporation were calculated by dividing the amount of reaction product by the reaction time. The *V*_max_ and *K*_*m*_ of nucleotide incorporation reactions were determined by nonlinear fitting of *V*_0_ as a function of dNTP concentrations, using GraphPad Prism 8 (GraphPad Software, Inc). The turnover numbers (*k*_cat_) were calculated by dividing *V*_max_ by molar number of Polκ. Each assay was performed thrice, independently.

### Molecular modeling

The coordinates and geometric constraints of GA-FAPy-dG were prepared using eLBOW of the Phenix suite (74) and built into the complex models by replacing the dG nucleotide opposite to the incorporating dCTP in the crystal structure of human Polκ in complex with the cisplatin DNA lesion (Protein Data Bank ID: 6BRX) (53). For the GA-FAPy-dG:thymidine base pair, the dCTP in the crystal structure was replaced with dTTP. The models were annealed by molecular dynamics simulations with phenix.dynamics for 1000 steps (500 fs) at 500 K and energy-minimized until convergence (100 steps at maximum) *in vacuo* with phenix.geometry_minimization using the Phenix suite. The donor–acceptor distances of the potential H-bonds (Fig. 7*B*) were restrained to 2.9 Å during the simulations. The potential energies of GA-FAPy-dG base pairs were evaluated using the CalcPotential function of MOE suite (75) with MMFF94x (54) and Amber14EHT (55) forcefields.

### Statistical analyses

The replication efficiencies and mutation frequencies of GA-FAPy-dfG in the TLS Pols KO cells were compared with their parental XP4PASV cells using an unpaired *t* test with Welch’s correction. Differences in the mutation spectrum of GA-FAPy-dfG in the aforementioned cells were evaluated using a Fisher’s exact probability test. *p* Values <0.05, calculated using two-sided tests, were considered statistically significant in all analyses. GraphPad Prism 8 was used for statistical analyses. Data are presented as mean ± standard error of the mean.

## Data availability

All the data described in this study are contained within the article.

## Supporting information

This article contains supporting information (76).

## Conflict of interest

The authors declare that they have no conflicts of interest with the contents of this article.
