# Supplementary material for: A formamidopyrimidine derivative from the deoxyguanosine adduct produced by food contaminant acrylamide induces DNA replication block and mutagenesis
Source: J Biol Chem. 2023 Jun 30;299(8):105002. doi: 10.1016/j.jbc.2023.105002 (PMC10406624; doi:10.1016/j.jbc.2023.105002)
Supplement: Supporting Figures S1–S6 and Tables S1 and S2 [file mmc1.pdf]

Supporting information for JBC_105002

A formamidopyrimidine derivative from the deoxyguanosine adduct produced by food contaminant acrylamide induces DNA replication block and mutagenesis

Jun-ichi Akagi, Masayuki Yokoi, Yumi Miyake, Tsuyoshi Shirai, Tomohiro Baba, Young-Man Cho, Fumio Hanaoka, Kaoru Sugasawa, Shigenori Iwai, Kumiko Ogawa

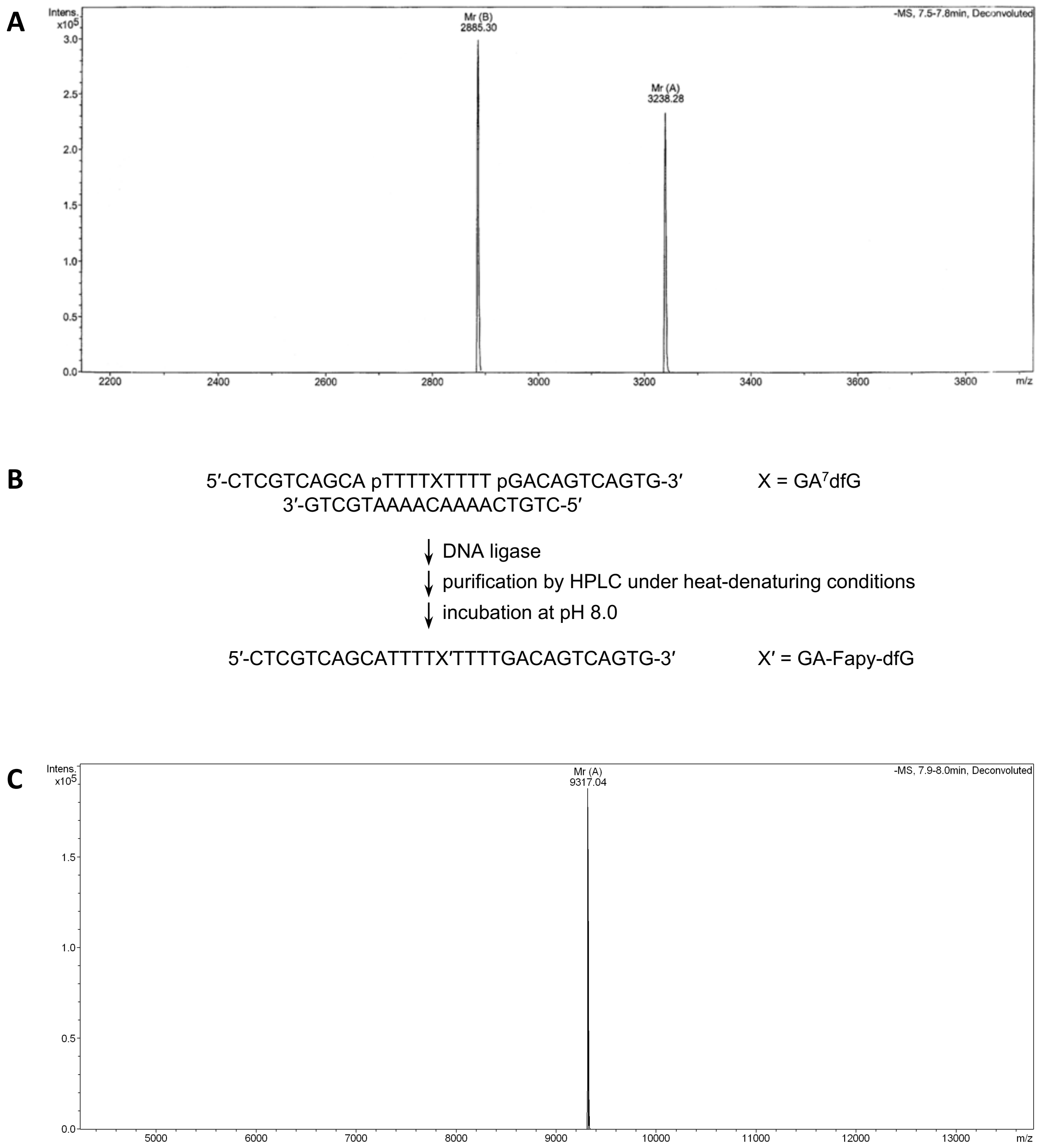

Figure S1. (**A**) ESI mass spectrum of the 9-mer, p-d(TTTTXTTTT) in which X is GA^{7}fG. Peak A is an internal standard, and the exact mass calculated for the desired product was 2885.46. (**B**) Preparation of the 30-mer template oligonucleotide containing GA-FAPy-dfG. (**C**) ESI mass spectrum of the 30-mer containing GA-FAPy-dfG. The *m*/*z* value calculated for the desired product was 9317.07.

A

single-stranded pMTEX-GA1 shuttle vector 5′-AGCCATCACTGACTGTCAAATTTAAATGCTGACGAGTCCGGA-3′

30-mer oligonucleotide carrying GA-adduct 3′-GTGACTGACAGTTTTXTTTTACGACTGCTC-5′

(X, the lesion site)

B

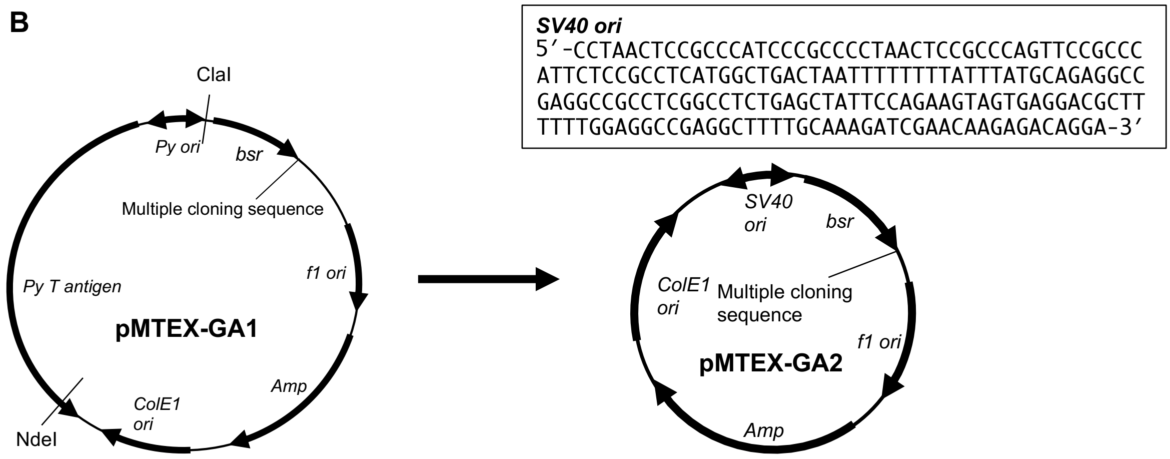

Figure S2. Construction of the pMTEX-GA vectors. (**A**) Multiple cloning sequence of a single-stranded pMTEX-GA1 shuttle vector complementary to the 30-mer oligonucleotide carrying 9-(2-deoxy-2-fluoro-β-D-arabinofuranosyl)guanine (GA[7]dfG) and with three-base mismatches opposite the lesion (underlined). (**B**) Polyoma (Py) virus origin (*ori*) and Py T antigen of the pMETX-GA1 vector were removed by NdeI and ClaI digestion. Chemically synthesized simian virus 40 (SV40) *ori* was ligated to create pMTEX-GA2. *Amp*, ampicillin resistance gene; *bsr*, blasticidin S resistance gene.

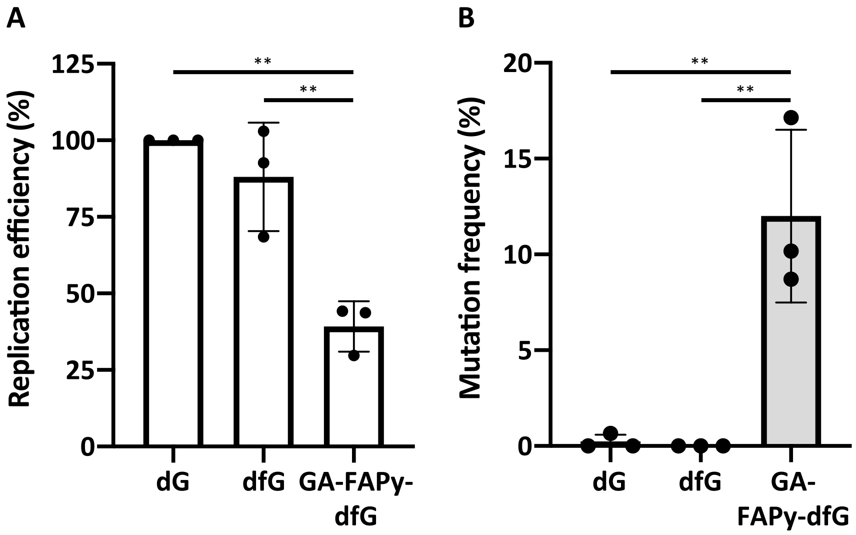

Figure S3. (**A**) TLS efficiency of GA-FAPy-dfG in XP4PASV cells expressed relative to the replication efficiency of the modified strand carrying dG. Tukey's multiple comparison test was used with $^{**}P < 0.01$. (**B**) Mutation frequency of the modified strand carrying dG, dfG, or GA-FAPy-dfG in XP4PASV cells. Tukey's multiple comparison test was used with $^{**}P < 0.01$.

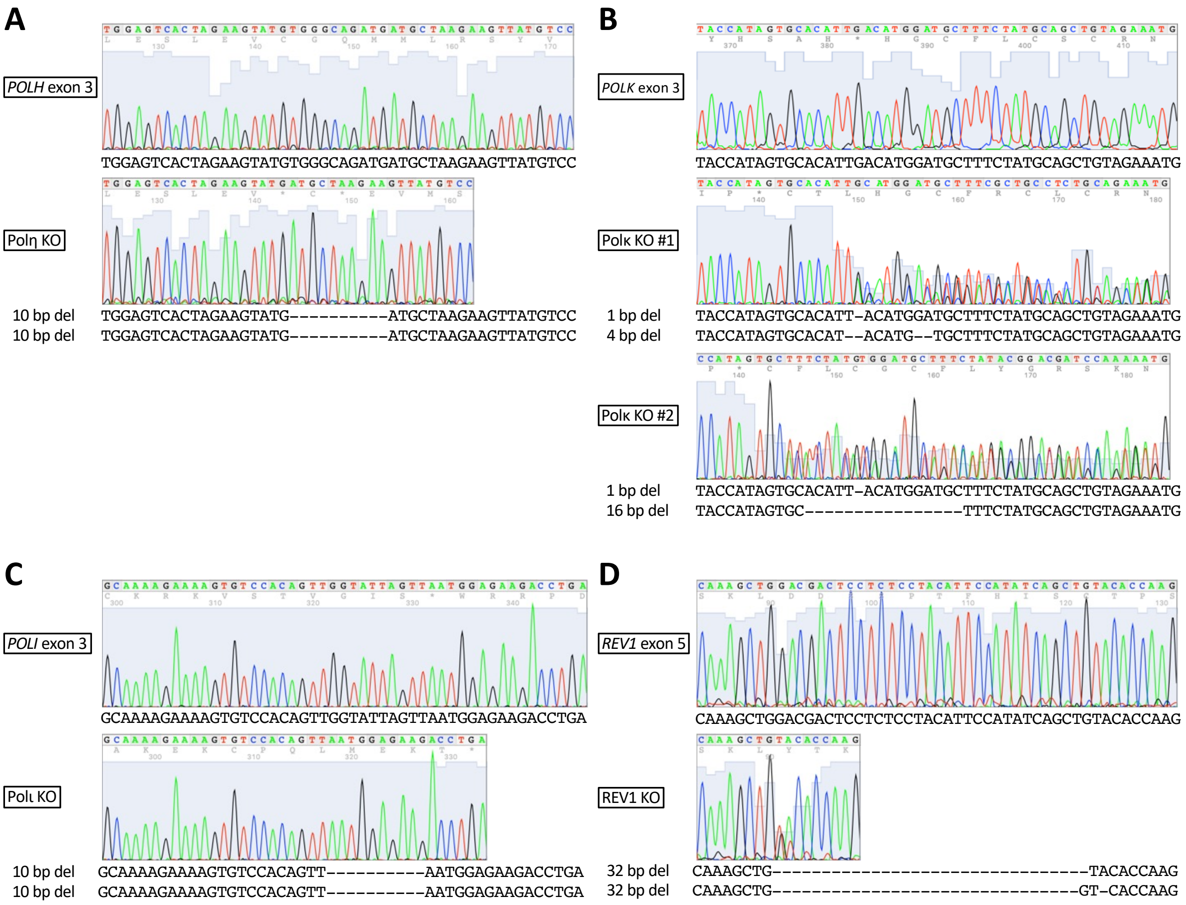

Figure S4. Sequencing analysis of target genes encoding Polη (*POLH*, **A**), Polκ (*POLK*, **B**), Polι (*POLI*, **C**), and REV1 (*REV1*, **D**) in the corresponding KO cells. For genomic sequencing, cells were lysed with 1% sodium dodecyl sulfate and genomic DNA was purified using QIAamp DNA Blood Mini QIAcube Kit (Qiagen). PCR was performed as follows: for *POLH* and *POLK*, an initial denaturing phase at 98°C for 2 min, 40 cycles at 98°C for 10 s, 60°C for 15 s, and 68°C for 30 s using MightyAmp DNA Pol V3 (TaKaRa) with primers {d(ATAGTTAGAACTACAGGAGCGC) [forward] and d(GAGACAAGATGGCTAACCAA) [reverse] for *POLH*; d(TATGTGCCTAAGGGTATGG) [forward] and d(GCAAACTGGTAGACCTCAAT) [reverse] for *POLK*}. For *POLI* and REV1, Platinum SuperFi II DNA Polymerase were used under the following conditions: an initial denaturing phase at 98°C for 30 s, a denaturing phase at 98°C for 5 s, and extension phase at 72°C for 15 s, and a final extension at 72°C for 5 min. For *POLI*, PCR was performed with an annealing phase of 5 cycles at 40°C for 10 s, followed by 35 cycles at 37°C for 10 s using primers {d(GGAGACAAAACAACTAGA) [forward] and d(CATCTTGACTTATGTTAT) [reverse]}. As PCR products were hardly detectable, the PCR reactants were purified and then the aliquots were further subjected to PCR twice. The second and third PCR were performed with annealing phases at 41°C and 40°C, respectively. For *REV1*, PCR was performed with an annealing phase of 35 cycles at 55°C for 10 s using primers {d(AGGACCACTAAAACCGAGCA) [forward] and d(CATACCATTGCACTCCAGCC) [reverse]}. The PCR products were subjected to sequencing analysis using following primers: d(TGAATGATGGCATCTGTGGT) for *POLH*; d(TATGTGCCTAAGGGTATGG) for for POLK; d(GGGTATGGAAAAATAATA) for *POLI*; d(GATGACCTGTTGTTGGTGAA) for *REV1*.

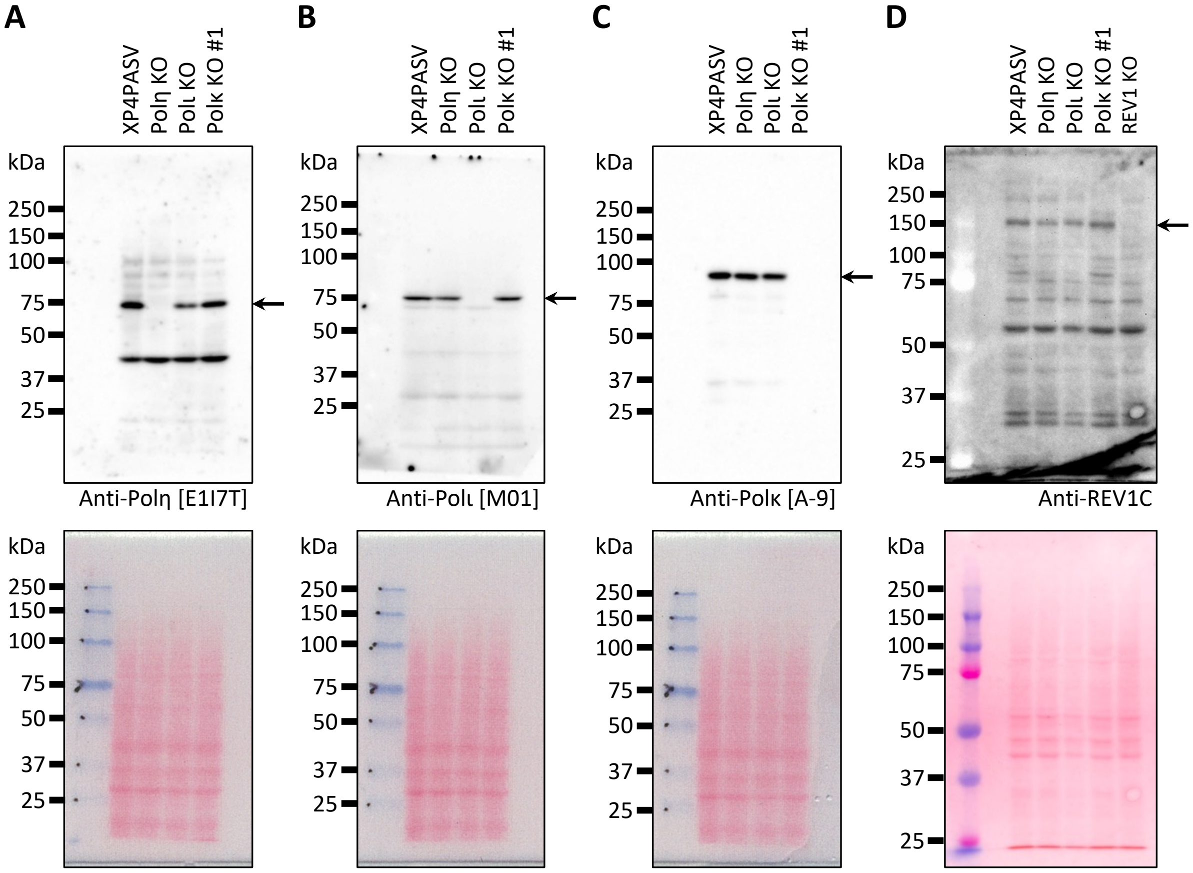

Figure S5. Western blot analysis of Polη (**A**), Polι (**B**), Polκ (**C**), and REV1 (**D**) KO cells. We subjected 10 μg protein of whole cell extract to sodium dodecyl sulfate-polyacrylamide gel electrophoresis and then transferred to poly(vinylidene fluoride) membrane. Proteins were detected using primary antibodies against Polη ([E1I7T], Cell Signaling Technology, Danvers, MA, USA), Polι ([M01], Abnova, Taipei, Taiwan), Polκ (DinB [A-9], Santa Cruz Biotechnology, Dallas, TX, USA), and REV1C (76) and corresponding horseradish peroxidase-conjugated secondary antibodies. Signals were detected by chemiluminescence using SuperSignal West Femto (Thermo Fisher Scientific; upper panels). The target proteins are indicated by arrows. Ponceau staining was used for loading control (lower panels).

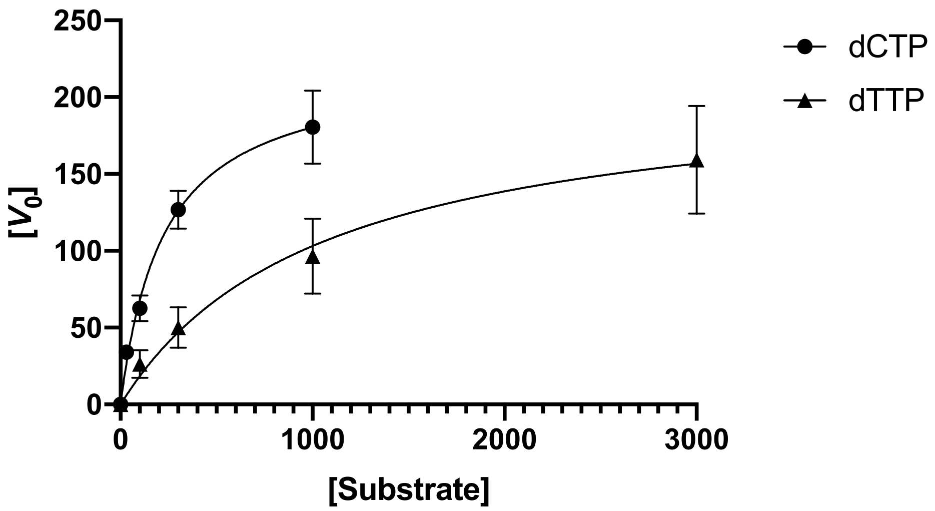

Figure S6. Michaelis–Menten plot of dCTP and dTTP incorporation opposite GA-FAPy-dfG by Polκ. Data are presented as the mean ± SEM.

Table S1. Mutation spectrum of GA-FAPy-dfG in TLS Pols KO cells

	XP4PASV		XP4PASV/*POLH*$^{-/-}$		XP4PASV/*POLK*$^{-/-}$ #1		XP4PASV/*POLK*$^{-/-}$ #2		XP4PASV/*POLI*$^{-/-}$		XP4PASV/*REV1*$^{-/-}$	
**Correct incorporation**	**289**	**(88.7)**	**163**	**(87.2)**	**189**	**(95.0)**	**166**	**(93.8)**	**115**	**(89.8)**	**158**	**(96.3)**
**Target misincorporation**	**32**	**(9.8)**	**24**	**(12.8)**	**7**	**(3.5)** *	**10**	**(5.6)**	**13**	**(10.2)**	**6**	**(3.7)** **
C̲ → A	6	(1.8)	3	(1.6)	2	(1.0)	5	(2.8)	2	(1.6)	4	(2.4)
C̲ → T	22	(6.7)	18	(9.6)	5	(2.5) *	5	(2.8)	9	(7.0)	0	(0.0) ***
C̲ → G	3	(0.9)	2	(1.1)	0	(0.0)	0	(0.0)	2	(1.6)	1	(0.6)
C̲ → Δ	1	(0.3)	1	(0.5)	0	(0.0)	0	(0.0)	0	(0.0)	1	(0.6)
**Semi-target misincorporation**†	**5**	**(1.5)**	**0**	**(0.0)**	**3**	**(1.5)**	**1**	**(0.6)**	**0**	**(0.0)**	**0**	**(0.0)**
5′-C̲A-3′ → 5′-CC-3′	1	(0.3)	0	(0.0)	1	(0.5)	0	(0.0)	0	(0.0)	0	(0.0)
5′-C̲A-3′ → 5′-CT-3′	0	(0.0)	0	(0.0)	1	(0.5)	0	(0.0)	0	(0.0)	0	(0.0)
5′-C̲A-3′ → 5′-CG-3′	0	(0.0)	0	(0.0)	1	(0.5)	0	(0.0)	0	(0.0)	0	(0.0)
5′-C̲AA-3′ → 5′-CAT-3′	4	(1.2)	0	(0.0)	0	(0.0)	1	(0.6)	0	(0.0)	0	(0.0)
**Total clones analyzed**	**326**	**(100.0)**	**187**	**(100.0)**	**199**	**(100.0)**	**177**	**(100.0)**	**128**	**(100.0)**	**164**	**(100.0)**

Mutation spectrum of GA-FAPy-dfG in TLS Pols KO cells compared with that in XP4PASV cells using the Fisher's exact probability test. $^{*}p < 0.05$, $^{**}p < 0.01$, $^{***}p < 0.001$. †Mutations within the three nucleotides next to the lesion. Underlining indicates the site of the lesion.

Table S2. Potential energies (kcal/mol) of base pairs with GA-FAPy-dG

Base pair	MMFF94x	Amber14EHT
dC:dG	−660.3 (−859.9)	−826.3 (−1090.0)
dC:*R*-GA-FAPy-dG	−316.6 (−725.3)	−516.9 (−934.4)
dC:*S*-GA-FAPy-dG	−300.2 (−724.9)	−510.6 (−934.6)
dT:*R*-GA-FAPy-dG	−317.8 (−767.0)	−546.5 (−972.6)
dT:*S*-GA-FAPy-dG	−307.3 (−764.8)	−540.4 (−974.3)

In parentheses are electrostatic potential components.

Supporting Information S1. A model of human Polκ in complex with dTTP and dsDNA containing GA-FAPy-dG in R-isomer (6brx_002fr.pdb). Available online at JBC website.

Supporting Information S2. A model of human Polκ in complex with dTTP and dsDNA containing GA-FAPy-dG in S-isomer (6brx_002fs.pdb). Available online at JBC website.

Supporting Information S3. A model of human Polκ in complex with dCTP and dsDNA containing GA-FAPy-dG in R-isomer (6brx_004fr.pdb). Available online at JBC website.

Supporting Information S4. A model of human Polκ in complex with dCTP and dsDNA containing GA-FAPy-dG in S-isomer (6brx_004fs.pdb). Available online at JBC website.
